# In Vitro and In Vivo Studies of Melanoma Cell Migration by Antagonistic Mimetics of Adhesion Molecule L1CAM

**DOI:** 10.3390/ijms25094811

**Published:** 2024-04-28

**Authors:** Stefano Vito Boccadamo Pompili, Sophia Fanzini, Melitta Schachner, Suzie Chen

**Affiliations:** 1 Department of Physiology and Pharmacology “V. Erspamer”, Sapienza University, 00185 Rome, Italy; 2 Susan Lehman Cullman Laboratory for Cancer Research, Ernest Mario School of Pharmacy, Rutgers University, Piscataway, NJ 08854, USA; sophiafanzini@gmail.com; 3 Department of Cell Biology and Neuroscience, Rutgers University, Piscataway, NJ 08854, USA; schachner@dls.rutgers.edu; 4 Rutgers Cancer Institute of New Jersey, New Brunswick, NJ 08901, USA; 5 VA New Jersey Health System, East Orange, NJ 07018, USA

**Keywords:** L1CAM, CD171, anagrelide, 2-hydroxy-5-fluoropirimidine, melanoma, cell migration in vitro, in vivo allograft

## Abstract

Melanoma, the deadliest type of skin cancer, has a high propensity to metastasize to other organs, including the brain, lymph nodes, lungs, and bones. While progress has been made in managing melanoma with targeted and immune therapies, many patients do not benefit from these current treatment modalities. Tumor cell migration is the initial step for invasion and metastasis. A better understanding of the molecular mechanisms underlying metastasis is crucial for developing therapeutic strategies for metastatic diseases, including melanoma. The cell adhesion molecule L1CAM (CD171, in short L1) is upregulated in many human cancers, enhancing tumor cell migration. Earlier studies showed that the small-molecule antagonistic mimetics of L1 suppress glioblastoma cell migration in vitro. This study aims to evaluate if L1 mimetic antagonists can inhibit melanoma cell migration in vitro and in vivo. We showed that two antagonistic mimetics of L1, anagrelide and 2-hydroxy-5-fluoropyrimidine (2H5F), reduced melanoma cell migration in vitro. In in vivo allograft studies, only 2H5F-treated female mice showed a decrease in tumor volume.

## 1. Introduction

Among cancers, metastatic melanoma is frequently resistant to standard therapies and has a poor prognosis. There are many risk factors associated with melanoma including UV exposure, fair skin, number of moles, and family history of skin cancer. Intermittent and chronic UV exposure from the sun or tanning beds induces many harmful effects, including an increase in reactive oxygen species (ROS). If the antioxidant capacity in cells does not balance with the increased ROS, the impact on various cellular functions and processes could enhance DNA damage and mutational burden. Melanoma being a ROS driven tumor has been suggested by several groups and remains to be a topic of intense study by many [1,2,3].

Small-molecule-based targeted therapies have resulted in dramatic initial responses, but these responses are usually short-lived, resistance emerges and relapse develops [4,5,6]. Immunotherapies have revolutionized the field of cancer treatment, but the complexity of tumor immune responses and immune evasion suggests that modulation of multiple immune-mediated pathways is necessary to further improve outcomes [7,8]. About half of metastatic melanoma patients develop brain metastases during their illness, making melanoma the disease with the highest propensity for dissemination to the central nervous system (CNS). There is an urgent need for the development of new approaches to treat patients afflicted with melanoma and melanoma metastasis. The migration of tumor cells is a prerequisite for tumor cell invasion and metastasis. Metastasis is a major cause of cancer-related death and occurs in a stepwise fashion, relying on several host–tumor interactions. To develop therapies focused on treating metastatic diseases, understanding the molecular mechanism of metastasis is vital. Therefore, we searched for a molecule that may play a role in melanoma metastasis. We focused on the cell adhesion molecule L1, which was discovered to be essential for the migration of neurons [9,10]. Expression of L1 is upregulated in many human cancers, including ovarian, pancreatic, melanoma, and glioblastoma [11,12,13]. In tumor cells, L1 promotes cell–cell adhesion in conjunction with proteolysis, conferring an invasive phenotype that supports aggressive tumor growth and metastasis [14,15]. Elevated levels of L1 expression in different cancer types correlate with poor prognosis, as shown by enhanced tumor cell proliferation, invasion, and metastatic dissemination [16,17]. In this scenario, L1 was suggested to be a biomarker and a driver in metastasis-initiating cells [18,19]. Taken together, these unique functional properties of L1 in cancer progression suggest that the inhibition of L1 may provide a strategy in the design of an anti-tumor metastasis-driven therapeutic approach. 

In the search for compounds that may reduce melanoma cell migration, we took advantage of the earlier studies in the identification of candidate small organic molecules by screening small organic compound libraries [12]. An ELISA competition approach was used to identify which compounds were L1 antagonistic mimetics. Finally, these compounds were tested for L1-dependent migration inhibition [12]. Three inhibitors of L1 functions that reduced the migration of glioblastoma cells in vitro were identified [12]. These three compounds displayed at least 50% interference with the L1 antibody binding to L1 and inhibited migration. The L1 antagonistic mimetics include anagrelide, 2-hydroxy-5-fluoropyrimidine (2H5F), and mestranol (excluded from the current study based on previous investigations by others [20,21,22,23]). We only used anagrelide and 2H5F in the current study. Anagrelide is a blood thinner used to treat thrombocythemia by reducing the platelet count to prevent megakaryocyte maturation in the bone marrow without interfering with other progenitor cells [24]. Also, 2-hydroxy-5-fluoropyrimidine (2H5F) is converted to 5-fluorouracil by hepatic aldehyde oxidase [25,26]; 5-fluorouracil inhibits metabolic processes in cells and is incorporated into RNA in tumor cells more often than normal cells. Adding 5-fluorouracil to RNA renders the RNA non-functional, thus reducing various tumor cellular functions [27,28]. As far as is known, no possible common overlapping pathways shared by 2H5F and anagrelide have been reported, except for the antagonistic mimetic L1 activity suppressing migration of various cancer cells [12]. U251 glioblastoma cells and various concentrations of each of these compounds were assessed in in vitro cell migration assays. Inhibition of U251 cell migration was shown at 1 μM for all three compounds; these findings were confirmed in the A172 cells, another glioblastoma cell line [12]. 

Based on the migration inhibitory activities of L1 antagonistic mimetics in glioma cells, we decided to examine if one or more of these compounds had the same effects in melanoma cells in vitro and in an animal model in vivo. We selected the melanoma model developed by our group, where we demonstrated an aberrantly expressed metabotropic glutamate receptor 1 (Grm1, mouse; GRM1, human) in the etiology of metastatic melanoma in transgenic [TG3 and Tg(Grm1)EPv] mouse models and subsequently showed its significance in the progression of human malignancy [29]. Stimulation of GRM1 results in the activation of two major signaling pathways critical in melanoma development/progression, MAPK and PI3K/AKT. This activation is independent of the most commonly mutated B-RAF and N-RAS genotypes in human melanoma [30]. Similar to human melanoma, our transgenic mouse models showed consistent metastases to various organs [31], suggesting Grm1-mediated melanocytic transformation may be a relevant model to investigate the function-suppressive potential of L1 antagonists in cell migration in vitro and in vivo. We found the antagonistic mimetics of L1 suppressed the migration of mouse melanoma cells but not cell proliferation in vitro. We detected a statistical significance in reduced allografted tumor only in female mice treated with 2H5F and no metastasis were noted.

## 2. Results

### 2.1. Isolation and Characterization of Mouse Melanoma Cells, MASS 3 with Luciferase Reporter

We performed a pilot study earlier with GFP-tagged human melanoma cells inoculated into five nude mice, and we detected lung metastases in all five mice and one brain metastasis (Chen et al., (unpublished data). The primary purpose of this pilot study was to assess the possibility of using the GFP reporter signal to monitor tumor progression and metastasis using the small animal imaging system, IVIS. We found that the GFP reporter was not sufficient to monitor or detect tumor metastasis in vivo using the small animal imaging IVIS system. We proceeded to the next approach, which was to isolate and use luciferase-tagged cells. 

**Figure 1:**
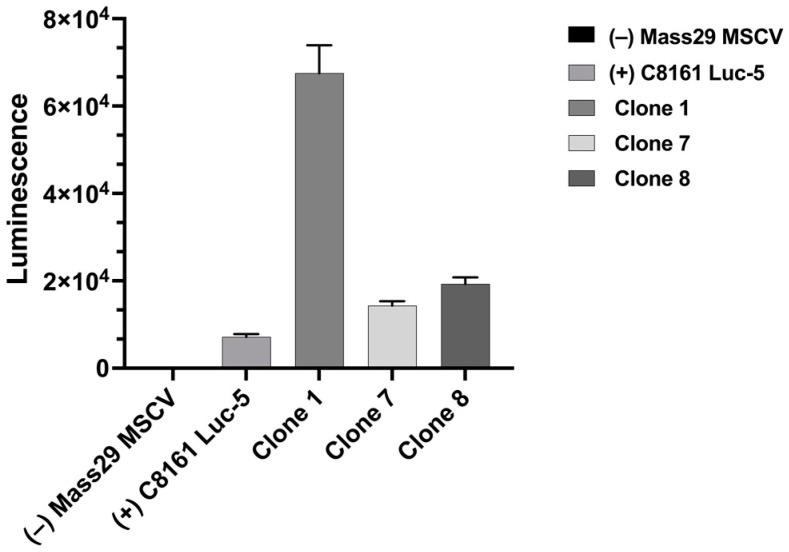
Several MASS 3 luciferase clones were isolated. Here is an example of the luminescence measurements on some MASS 3 luciferase-tagged clones. MASS 29 MSCV, a negative control clone, was infected with an empty lentivirus vector, C8161 Luc 5, a positive control. Clones 1, 7, and 8 are independent MASS 3 luciferase-tagged clones. Data are expressed as Luminescence ± SEM.

**Figure 2:**
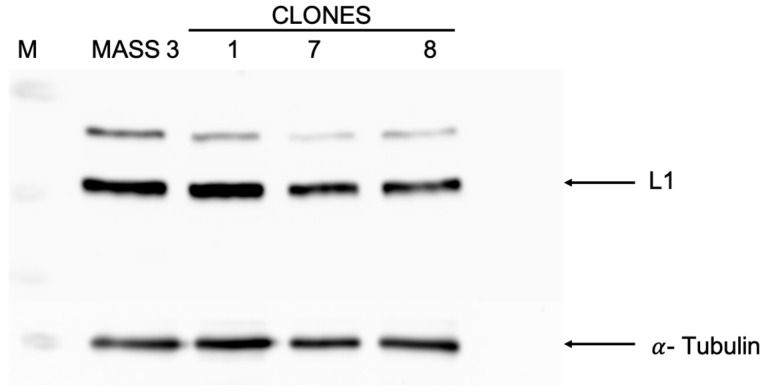
Western immunoblot of MASS 3 parent and three clones with luciferase reporter (M = marker, parent, luciferase clones 1, 7 and 8); α-Tubulin was used as a loading control.

MASS 3 cells were infected with a lentiviral vector carrying the luciferase reporter gene. We isolated 30 clones and performed luminescence measurements with a Tecan Infinite 200 Pro plate reader. An example is shown in Figure 1. MASS 3 clone 1 was selected for the current study. We performed Western immunoblots with protein lysate prepared from several MASS 3 clones that showed luciferase activities (Figure 1). We detected L1 expression in all samples tested (Figure 2).

### 2.2. L1 Antagonistic Mimetics Reduced Migration of Mouse Melanoma Cells

**Figure 3:**
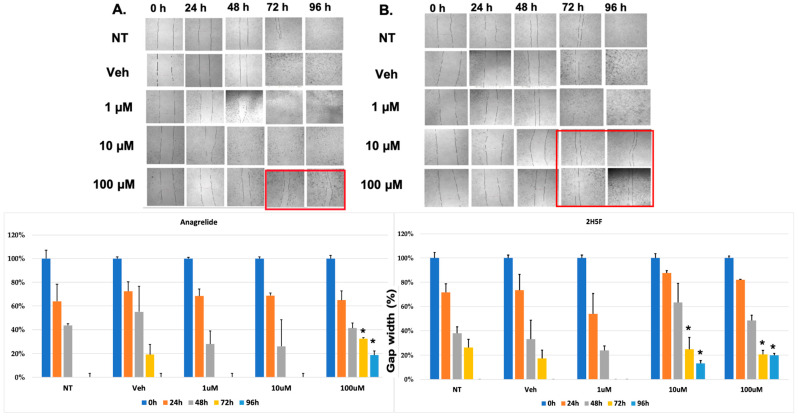
Scratch assays were performed with mouse melanoma MASS 3 clone 1 cells, NT (no treatment), Veh (DMSO, 0.5%), (**A**) anagrelide or (**B**) 2H5F at three different concentrations (1, 10, and 100 μM) for 24, 48, 72, and 96 h. Images were acquired every 24 h. Quantification of the gap for each timepoint and treatment is the average of three acquired images and expressed as percentage ± SEM. The comparison was between vehicle and treatment group at respective time point, i.e., the 24 h treatment was compared with 24 h veh. Two-way ANOVA was used to determine statistical significance, *, *p* ≤ 0.05. Red frames denoted significant differences when compared to controls.

The scratch assay is a simple in vitro assay to quantify cell migration over time with different treatment modalities. We performed preliminary migration assays with luciferase-tagged MASS 3 clone 1 cells and used two L1 antagonistic mimetics, anagrelide and 2-hydroxy-5-fluoropyrimidine (2H5F). Anagrelide at 100 μM showed the most inhibition in cell migration at 72 and 96 h (Figure 3A and Figure S1); 2H5F at 10 μM and 100 μM showed suppression of cell migration at 72 and 96 h (Figure 3B and Figure S1). Taken together, these results demonstrated that antagonistic mimetics of L1 are able to reduce mouse melanoma cell migration in vitro; furthermore, we concluded that 2H5F appears to be more effective than anagrelide. 

### 2.3. Cell Viability and Cell Proliferation MTT Assay

**Figure 4:**
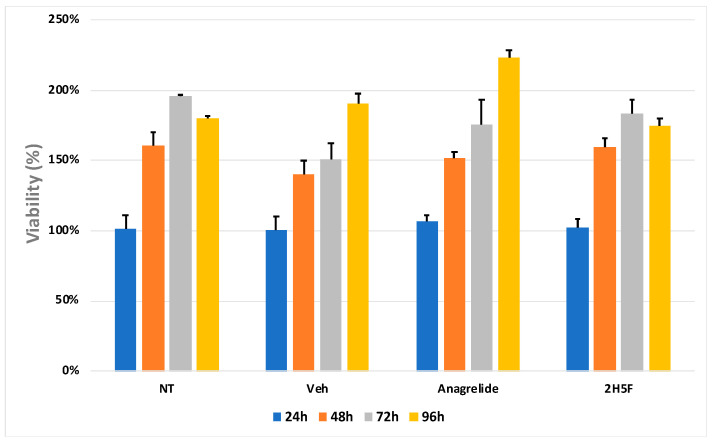
MTT assays were performed with MASS 3 clone 1 cells for 24, 48, 72, and 96 h, NT (no treatment), Veh (DMSO 0.5%), anagrelide (100 μM), or 2H5F (100 μM). The vehicle values at each time point (24, 48, 72, and 96 h) were normalized to itself (100%), and the values from the treated samples were normalized to the vehicle that corresponded to the same time point. Data are expressed as percentage ± SEM from three independent experiments. No statistical significance was found.

We then performed MTT assays to examine if one or both antagonistic mimetics of L1 may influence cell proliferation and viability in mouse melanoma cells. No effect was observed after 96 h of treatment (Figure 4). These results were in line with previous studies involving glioblastoma cells [12]. The cell growth MTT assays showed that neither 2H5F nor anagrelide modulated mouse melanoma cell viability or proliferation. 

### 2.4. No Alterations in L1 Expression in Luciferase-Tagged MASS 3 Clone 1 Melanoma Cells 

**Figure 5:**
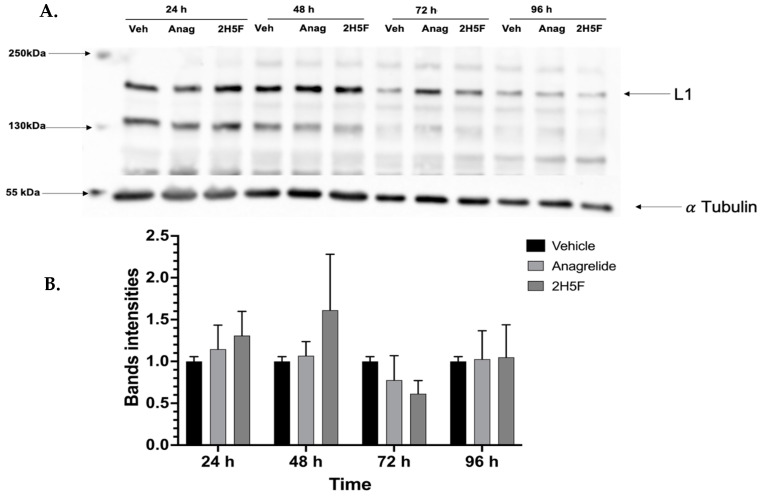
(**A**). Representative Western immunoblot of L1 in MASS 3 clone 1 cells treated with vehicle (DMSO, 0.5%), anagrelide (100 μM), or 2H5F (100 μM) for 24, 48, 72, and 96 h. Loading control was α-Tubulin. (**B**). Quantification of the protein band intensities corresponding to each lane from three independent experiments were used. Bands are represented as mean ± SEM, vehicle vs each treatment group, no statistical significance was found.

We then investigated the presence of the antagonistic mimetics modulated L1 expression in our mouse melanoma cell line. We prepared protein lysates from MASS 3 clone 1 cells treated under the same conditions as the MTT assay (Figure 4) and performed Western immunoblots. Three independent Westerns were performed and an example is shown (Figure 5A). L1 expression was detected in all samples (Figure 5A). We quantified L1 expression levels and normalized to vehicle treatment at the respective timepoints using values from all three independent Westerns, no significant differences were detected (Figure 5B). 

### 2.5. In Vivo Assessments of Allografts by Vernier Caliper and IVIS

**Figure 6:**
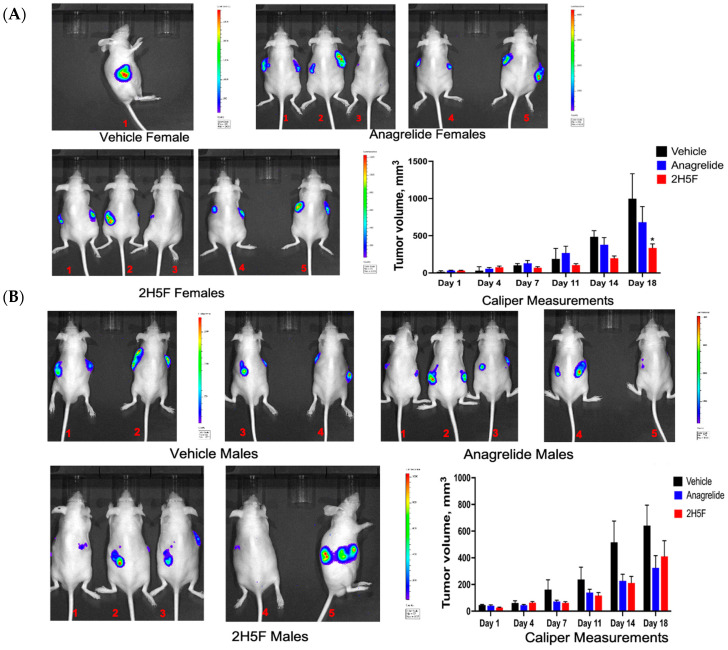
Five males and five females were used in vehicle and each treatment group. A representative image is shown for each group of hairless C57BL/6 mice with inoculated MASS 3 clone 1 tumor cells. (**A**) Female mice were treated with vehicle (DMSO), anagrelide (20 mg/Kg), or 2H5F (10 mg/Kg) (**B**) Male mice, same treatment modalities as the female mice. Two-way ANOVA with Bonferroni post hoc correction ± SEM, *, *p* ≤ 0.05; vehicle vs. treatment.

Five male and five female mice were used in each group, vehicle, treatment with 2H5F or anagrelide. MASS 3 clone 1 cells (10^6^) were inoculated into both flanks of immunocompetent hairless C57BL/6 [32]. Palpable tumors appeared after 10 days. The tumor volumes were measured twice a week with a vernier caliper and the small animal imaging IVIS system was used to detect possible metastasis (Figure 6). When the primary tumors reached ~700 mm^3^, we performed survival surgeries to remove the tumors and continued to monitor for tumor migration/metastasis. Based on the caliper measurements, it appeared that primary tumors in 2H5F-treated female hairless C57BL/6 mice showed statistically smaller tumor volumes compared to vehicle or anagrelide treated mice after 18 days of treatment; a representative image is shown for each group (Figure 6A). In contrast, male mice did not show any significant difference in tumor volume regardless of treatment (Figure 6B). Secondary/tertiary tumors appeared after several weeks (ranged from 4–6 weeks), almost always near the primary tumor site, suggesting the survival surgeries did not completely remove all tumor cells. We did not compare the secondary/tertiary tumor volumes between vehicle and treatment groups because we cannot be sure how many cells were left behind from the surgeries. 

### 2.6. Expression of L1 in Excised Tumors

**Figure 7:**
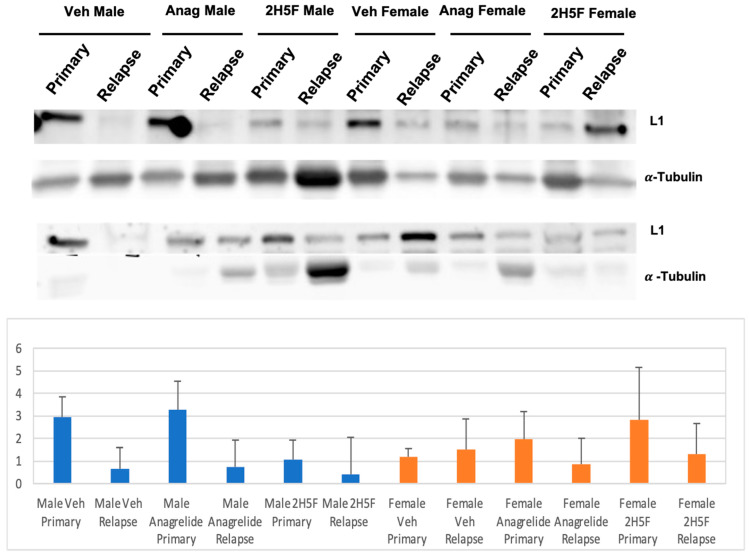
Western immunoblots of L1 expression with protein lysates prepared from excised tumor samples in different treatment groups, vehicle (DMSO), anagrelide (20 mg/Kg), or 2H5F (10 mg/Kg). Loading control was α-Tubulin. The loading order was the same in both Western blots shown ((**top**,**bottom**) panels). Protein band intensities were normalized to loading control. Data are expressed as intensity mean ± SEM. No statistical significance was found.

When the primary tumors reached a volume of 700 mm^3^, we performed survival surgeries to remove the tumors and then continued to monitor tumor progression. We performed Western immunoblot analyses with protein lysates prepared from excised tumors and compared for possible differences in L1 expression levels between primary and relapsed secondary tumors. Two examples of Western blots for measuring L1 levels and loading control of α-Tubulin are shown below (Figure 7). The Western blots show the same lane order, but with different tumor protein lysates prepared from various mice. For example, lane 1 in the top panel contains lysates from a primary tumor of a vehicle-treated male mouse, while lane 1 in the bottom panel has lysates from a different vehicle-treated male mouse’s primary tumor. The quantification scans shown were values obtained from three independent experiments (Figure 7). In all cases, except for the female vehicle treated group, L1 levels appeared to be lower in primary tumor samples compared to relapse secondary tumor samples, but none of the comparisons showed statistical significance.

## 3. Discussion

Despite accounting for only 1% of diagnosed skin cancer cases, melanoma is responsible for the majority of deaths attributed to skin cancer each year. In the United States, about 100,000 new cases of invasive melanoma and over 8000 fatalities are predicted in 2024 [33,34]. Much progress has been made in the past 10–15 years with targeted therapies against mutated BRAF, the most common mutation in cutaneous melanoma, and immune-checkpoint blockade immunotherapies [35,36,37,38,39]. However, melanoma remains one of the most difficult cancers to treat, with a high frequency of relapse and resistance, suggesting that a better understanding of melanoma biology is still needed.

In this study, we explored the role of an adhesion molecule, L1, in melanoma cell migration. Earlier studies demonstrated that overexpression of L1 is involved in tumor development, tumor cell invasion, and metastasis of melanoma, ovarian, and colon cancer [40]. L1 was shown to promote malignant cell mobility and is linked to the activation of multiple signaling pathways known to be critical in tumor cell proliferation and survival, including extracellular signal-regulated kinase (ERK), focal adhesion kinase (FAK), and p21-activated kinase (PAK) [41,42,43]. Two mimetic antagonists of L1, anagrelide and 2H5F, were used in the present study, and showed a reduction in mouse melanoma cell migration, but not cell proliferation in vitro. In vivo tumorigenicity assays showed that 2H5F is effective in reducing tumor volume, significantly in female mice, compared with vehicle control as early as after 14 days of treatment. Unfortunately, we did not observe any metastasis development, contrary to our earlier pilot study with human C8161 cells [44]. One difference could be that the C8161 human melanoma cells were established from a metastatic tumor while the MASS cells we used in the current study were derived from in vitro selection of transformed cells after transfection with Grm1 cDNA. The differences between the human C8161 and mouse MASS cells may be extensive with respect to various parameters in cell growth and interactions with the surrounding microenvironments, plus some intrinsic variations in human and mouse cells. We have an ongoing unrelated in vivo study using the same MASS cells tagged with luciferase and we observed reduced luminescence signals over time in vivo, which suggested that it is not feasible to use these MASS cells to monitor for metastasis by IVIS. When we terminated the current study, we did not detect metastasis by IVIS or visually during necropsy in any organs. Taken together, our results showed that both antagonistic mimetics of L1 reduced the melanoma cell migration in vitro but not in vivo. The observation that 2H5F-treated female mice displayed a reduction in tumor volumes agrees with other gender biased responses to therapeutic treatment that have been observed by others [45]. This sex-linked dichotomy in treatment responses is a contentious issue, with sex hormones playing a role but contradictory data suggesting other factors. The female immune system’s benefits may explain gender differences, but recent immunotherapies show greater results in men [46,47,48].

Sex differences are caused by a variety of mechanisms, including sex steroid hormone-dependent and independent factors. These elements work on several levels, from the tumor to the organs that influence tumor growth, such as the immune system [49,50].

According to epidemiological studies, women have an advantage over men in terms of melanoma incidence, progression, and survival [51], and in the work of Dakup and colleagues they observe that over 14 days, female mice had a considerably smaller tumor volume and growth rate than male mice, as we observe. To date, we know that anagrelide is an approved drug to treat coagulation diseases, such as essential thrombocythemia, but we only found one report that described small differences between males and females [52]. No sex differences in humans were reported for 2H5F. Thus, the difference observed between the sexes in vivo does not have an unambiguous and definite explanation, but confirms the active ongoing debate about the possible causes of gender differences in melanoma and other tumor occurrence.

## 4. Materials and Methods

### 4.1. Selection of Luciferase-Tagged Mouse Melanoma Cell Lines 

Mouse melanoma MASS clones were made by introducing Grm1 cDNA under the melanocyte-specific promotor, dopachrome tautomerase, DCT, in immortalized non-tumorigenic mouse MelanA cells derived from C57BL/6 mice. Several independent MASS clones were isolated and shown to be transformed in vitro and tumorigenic in vivo [53]. In the current study, we selected MASS 3 cells and infected the cells with a lentiviral vector containing luciferase, pLentipuro3/TO/V5-GW/EGFP-Firefly Luciferase, which was a gift from Ethan Abel (Addgene plasmid #119816; http://n2t.net/addgene:119816; RRID: Addgene_119816). Several independent MASS 3-Luc clones were isolated and characterized further with puromycin added in the medium as the selection agent. Cells were grown in 60 mm Petri dishes (Sigma, St. Louis, MO, USA) in complete selection medium (RPMI 1640 with 10% FBS, 100 U/mL-100 μg/mL penicillin-streptomycin, 0.8 mg/mL puromycin, Sigma, St. Louis, MO, USA) to 85% confluence for all experiments. 

### 4.2. Luminescence Assays 

The luminescence assays were used to confirm the luciferase reporter in MASS 3 clones. We used “Dual-Luciferase® Reporter Assay System” from Promega (Promega Corporation, Madison, WI, USA). The test was performed following the protocol provided by the manufacturer. 

### 4.3. Protein Lysate Preparations from Mouse Melanoma Cell Lines

The cells were washed twice with cold 1× PBS, pH 7.3, Laemmli Sample Buffer (100 μL per 60 mm plate, Bio-Rad Laboratories Inc. Philadelphia, PA, USA, Cat. # 1610747) plus 5% β-mercaptoethanol (Sigma, St. Louis, MO, USA) was added. Using a cell scraper, the mixture with the cells was collected, heated at 99 °C for 10 min, and centrifuged at 18,407× *g* for 10 min; the supernatants were stored at −80 °C for future investigation.

### 4.4. In Vivo Tumor Lysate Preparations

Tumors were excised from the mouse, snap-frozen in liquid nitrogen and stored at −80 °C until the preparation of lysates. Lysis buffer consisted of 50 mM Tris HCl, 150 mM NaCl, 1 mM EDTA, 5% Glycerol, 1% Igepal at pH 7.5, DTT 1 mM, cocktail phosphatase 3 inhibitors (Sigma, St. Louis, MO, USA, Cat. #P0044), and cocktail phosphatase 2 inhibitors (Sigma, St. Louis, MO, USA, Cat. #P5726), and 25× proteinase inhibitors (Sigma, St. Louis, MO, USA, Complete Mini-EDTA-free, 11836170001) were added to the lysis buffer to 1× concentration before the extraction procedure. Pestles and mortars were pre-cooled with liquid nitrogen, each tumor sample was added, crushed, and reduced to powder using the pestle, then liquid nitrogen was added occasionally to maintain the cold temperature. Once a fine powder was obtained, the contents were poured into a sterile polypropylene tube and 250 μL of lysis buffer was added. A polytron (OMNI-TH International, Kennesaw, GA, Polytron) was used to further break down the tissue samples. The mixture was placed on a shaker at 4 °C for 2 h. The sample was then transferred to a fresh tube and centrifuged at 18,407× *g* at 4 °C for 20 min. The supernatant was recovered and placed in a new tube and stored at −80 °C for further analyses.

### 4.5. Western Immunoblots

Due to the pigmentation in in vivo excised tumor samples, protein concentration cannot be reliably determined and, thus, was not performed; instead, we included loading controls in all Westerns, 30 μL of protein extracts (from cells or tissue samples) were loaded onto 7.5% SDS-PAGE gels and then separated by electrophoresis at 100 volts for approximately 2 h. Proteins were then transferred onto a nitrocellulose membrane at 160 mVolts for 4 h at 4 °C. The membrane was stained with Ponceau Red to make sure the transfer was uniform before incubation with 5% non-fat dry milk in Tris-buffered saline with 0.1% Tween20 detergent (TBST) for 1 h at room temperature. Membranes were incubated with the following primary and secondary antibodies prepared in 0.25% non-fat dry milk in TBST. Primary anti-L1 rabbit polyclonal antibody (Sigma, St. Louis, MO, USA, Cat. #SAB4501674) was used at 1:1000; primary tubulin antibody (monoclonal anti-α-tubulin, Sigma, St. Louis, MO, USA, Cat. #T6074) was used at 1:10,000. Secondary anti-rabbit antibody for L1 (EMD Millipore, Burlington, MA, USA, Cat. #AP182P) or anti-mouse secondary antibody for tubulin (Sigma, St. Louis, MO, USA, anti-mouse IgG, Cat. #A4416) was used at 1:5000.

### 4.6. MTT Cell Viability/Proliferation Assay

Mouse melanoma cells were seeded in a 96-well plate at a concentration of 1 × 10^4^ cells per well in 50 µL in complete RPMI-1640 medium and incubated for 24 h. After 24 h, anagrelide or 2H5F was added to a final concentration of 100 µM in 50 µL, and readings were taken after 24, 48, 72, and 96 h using a plate reader at a wavelength of 565 nm and a reference wavelength of 750 nm (Tecan, Männedorf, Kanton of Zürich, Switzerland, Infinite 200 Pro plate reader). Anagrelide (CAS #68475-42-3) and 2-hydroxy-5-fluoropirimidine (CAS #2022-78-8) were purchased from Sigma (St. Louis, MO, USA). The data reported are derived from three independent experiments; vehicle values at each time point (24, 48, 72, and 96 h) were normalized to itself to yield 1 (100%), and the values from the treated samples were normalized to the vehicle corresponding to the same time point, i.e., 24 h treatment normalized to 24 h vehicle.

### 4.7. Migration Scratch Assays

Mouse melanoma cells were seeded at 2 × 10^5^ cells per well in a 6-well plate and maintained for 24 h; after 24 h, the tip of a 200 μL pipette was used to create a scratch along the entire length of the well. The wells were then washed twice with sterile PBS and maintained in culture medium (RPMI 1640 with 10% FBS and 100U/Ml–100μg/mL penicillin-streptomycin, 0.8 mg/mL puromycin (Sigma, St. Louis, MO, USA). The four treatment groups were: no treatment (NT); DMSO (Vehicle, 0.5%); anagrelide (1 μM, 10 μM, or 100 μM); or 2H5F (1 μM, 10 μM or 100 μM). The medium was replaced with fresh medium containing the corresponding reagents after 48 h. Images were acquired every 24 h using a Nikon Eclipse Ti-U microscope, Nikon DS-Qi2 camera, and NIS-elements software (NIS-elements BR 5.11.00 64-bit). All data are normalized against the respective vehicle at corresponding time points, i.e., 24 h treatment normalized to 24 h vehicle.

### 4.8. In Vivo Allografts

For the in vivo studies, 5 female and 5 male BL6/SKH (hairless C57BL/6) [32] mice at 8–10 weeks of age were used for vehicle and each of the treatment groups. The animals were bred and maintained at our Association for Assessment and Accreditation of Laboratory Animal Care (AAALAC) accredited facility. The animals were housed in controlled environmental conditions: temperature 20 ± 2 °C, relative humidity 50 ± 10%, ventilation with 15 spare parts air hour, and regular day/night cycle (12 light/12 dark) with water and food *ad libitum*. All experiments were carried out in compliance with Rutgers Institutional Animal Care and Use Committees (IACUCs). Mouse melanoma cell lines, 1 × 10^6^ MASS 3 clone 1 luciferase-tagged cells resuspended in PBS were inoculated by subcutaneous injection in a volume of 100 μL (50 μL cells and 50 μL Matrigel) in each flank. When the tumor became palpable, the animals were randomly divided into three treatment groups: vehicle (DMSO diluted in sterile PBS), anagrelide (20 mg/kg), and 2H5F (10 mg/kg); the treatments were administered three times a week by intraperitoneal injection. Tumors were measured twice a week with a two-dimensional vernier caliper and once a week by the small animal imaging IVIS system. The animals were sacrificed at the end of the treatment, or at the first sign of distress. To determine the minimum statistically valid number of mice to use per group, we consulted Rutgers Biometrics Facility Core. Under their guidance, we used a regression model designed to detect at least a 35% inhibition of tumor growth using an assumption of a standard deviation of 25% of the value of the means for the experimental groups. For estimating the power/sample size, we assume, based on preliminary results, that the standard deviation of each group will be approximately 25% of the mean of the group at the end of the experiment. With 5 male and 5 female mice per group, the detectable difference is 50%. Mixed effect models for repeated measurements were used for the analysis of the average tumor lesion size (tumor burden) over time.

### 4.9. Measurement of Tumor Volume with Vernier Caliper In Vivo

Measurements of tumor volumes by digital vernier caliper were performed twice a week. The tumor volume, in mm^3^, was calculated as (A × B^2^)/2; A and B are length and width of the tumor, respectively. The vernier caliper used was Fowler (Veterinary Instrumentation, Sheffield, UK, Tools and Instruments, ultra-cal IV 6”/150 mm).

### 4.10. In Vivo Imaging Using the Smalll Animal Imaging IVIS System 

To visualize the luciferase reporter in the mouse melanoma cells, MASS 3 clone 1, we prepared 100 mg of Luciferine Potassium Salt (Regis, Technologies Inc., Morton Grove, IL, USA, Code 1-360223-200) in 6.6 mL of sterile 1X PBS to obtain a concentration of 15 mg/mL, and 200 μL of the solution was injected intraperitoneally into each mouse at least 15 min before acquisition of images from the small animal imaging system, IVIS (IVIS^®^ imaging system, Perkin-Elmer, Inc., Shelton, CT, USA). We used the intensity of the emitted bioluminescence from the luciferase reporter in MASS 3 clone 1 cells to monitor the tumor growth, tumor cell migration, and possible metastasis development.

### 4.11. Statistical Analysis

Appropriate sample sizes for animal studies were determined in consultation with the Rutgers Biometrics Facility Core. Statistical significance was determined using StatPlus Pro version 6 from the AnalystSoft, Inc. (Brandon, FL, USA) plug-in, in Microsoft Excel (Microsoft Excel software version 16051.17425.20176.0). Analyses were conducted using two-way ANOVA test with two variables (gender and treatment modality) being tested and Bonferroni post-hoc analyses were performed to determine statistical significance between treated pairs, *p* ≤ 0.05 is considered significant. To quantify immunoblots we used ImageJ software version 1.52. In statistical analyses we used the vehicle group for comparisons with the treatment groups.

## Data Availability

Not applicable.

## Supplementary Materials

The following supporting information can be downloaded at: https://www.mdpi.com/article/10.3390/ijms25094811/s1.
