# Supplementary material for: In Vitro and In Vivo Studies of Melanoma Cell Migration by Antagonistic Mimetics of Adhesion Molecule L1CAM
Source: Int J Mol Sci. 2024 Apr 28;25(9):4811. doi: 10.3390/ijms25094811 (PMC11084881; doi:10.3390/ijms25094811)

Scratch assay figures

Supplementary material 1

**Microscope**	**Nikon Eclipse Ti-U**
**Camera**	**Nikon DS-Qi2**
**Acquisition Software**	**NIS-elements**
**Microscope magnification**	**4 X**
**Time points**	**0, 24, 48, 72, 96 hours**
**Sperimental Groups**	**No Treatment (NT)**<br>**Vehicle (DMSO)**<br>**Anagrelide (1, 10 and 100 µM)**<br>**2H5F (1, 10 and 100 µM)**
**Scale**	**µm**

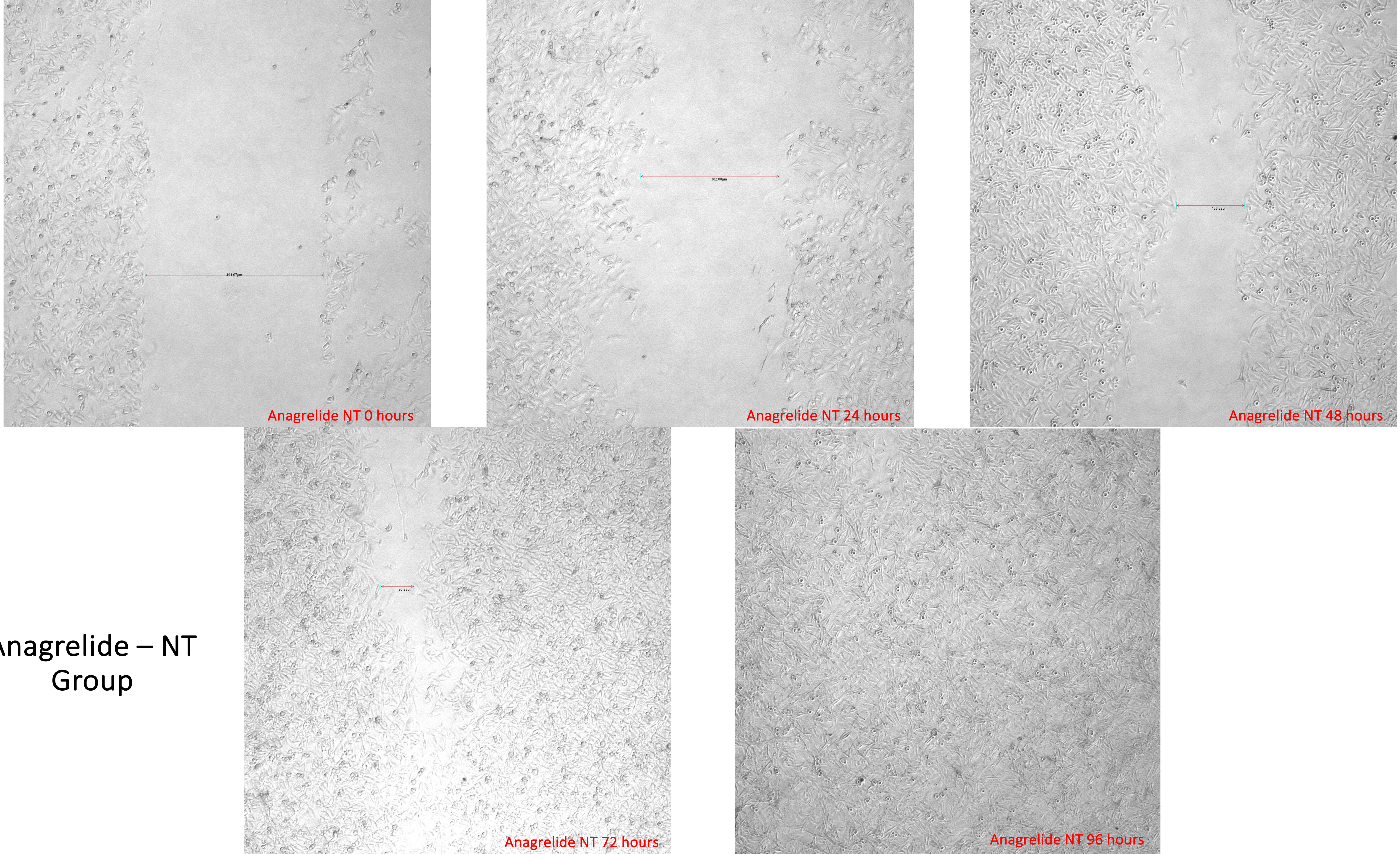

Anagrelide – NT Group

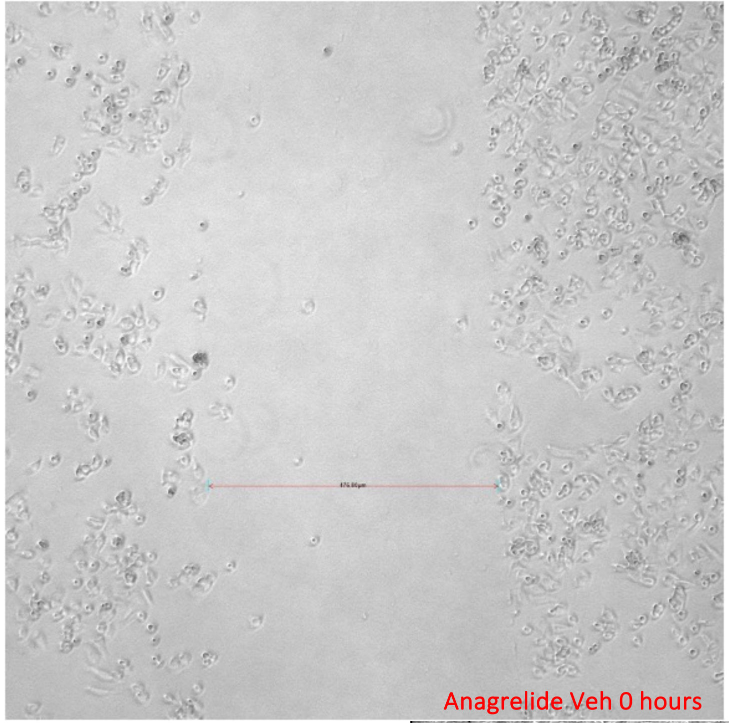

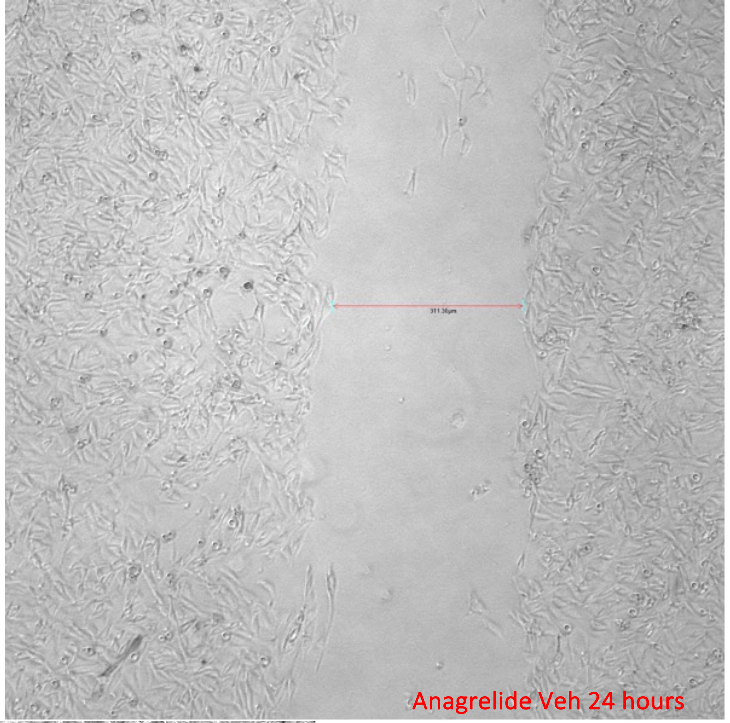

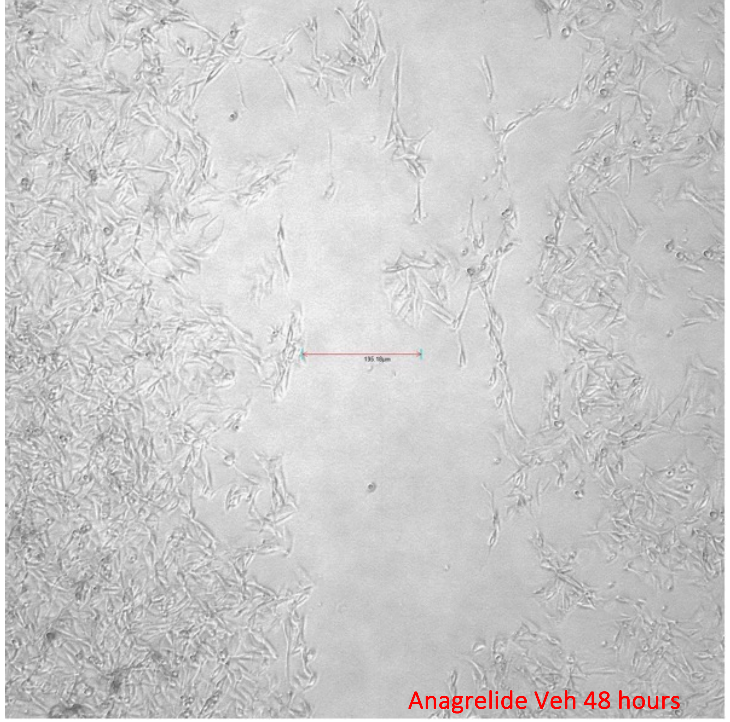

Anagrelide – Veh Group

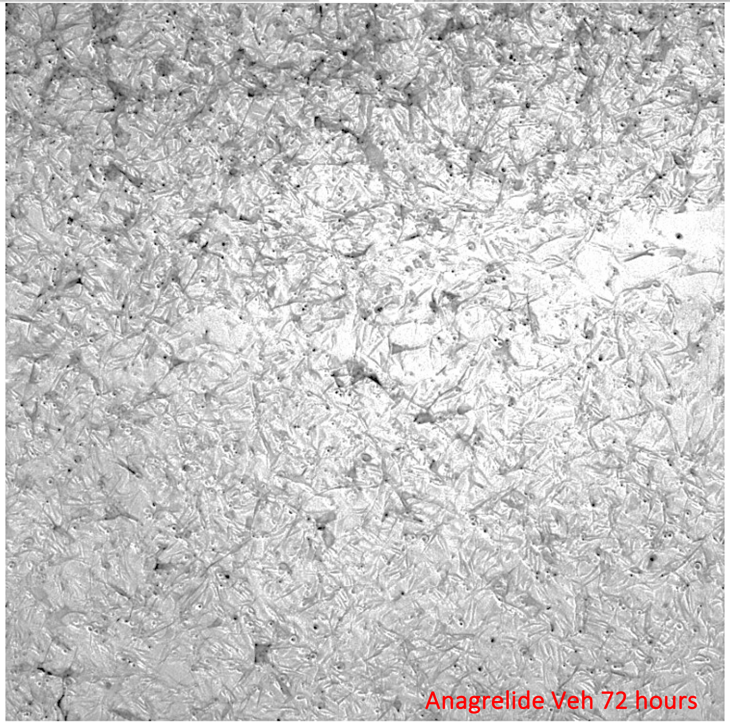

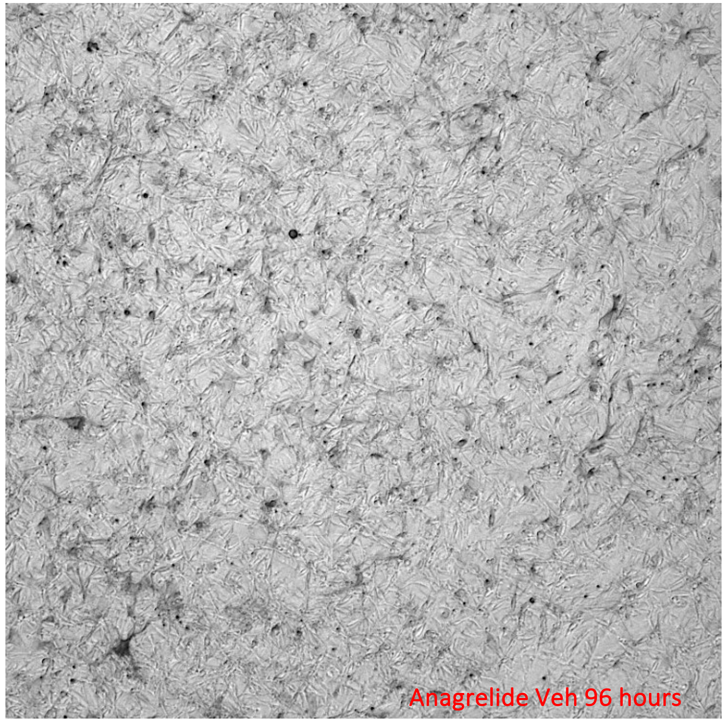

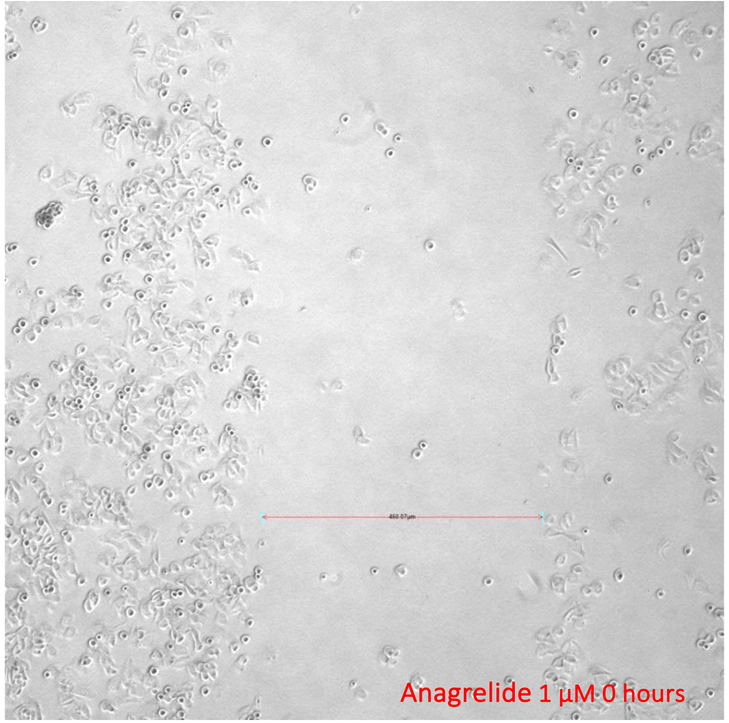

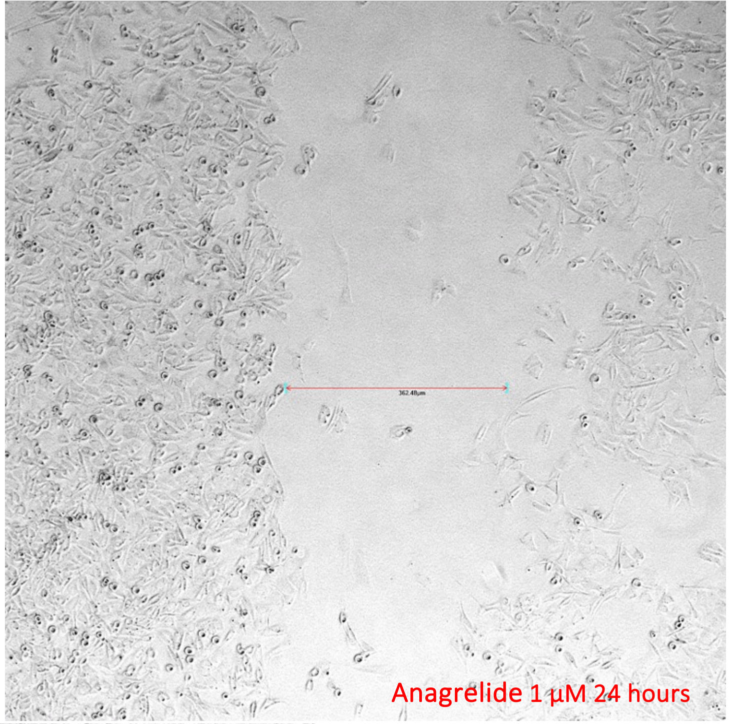

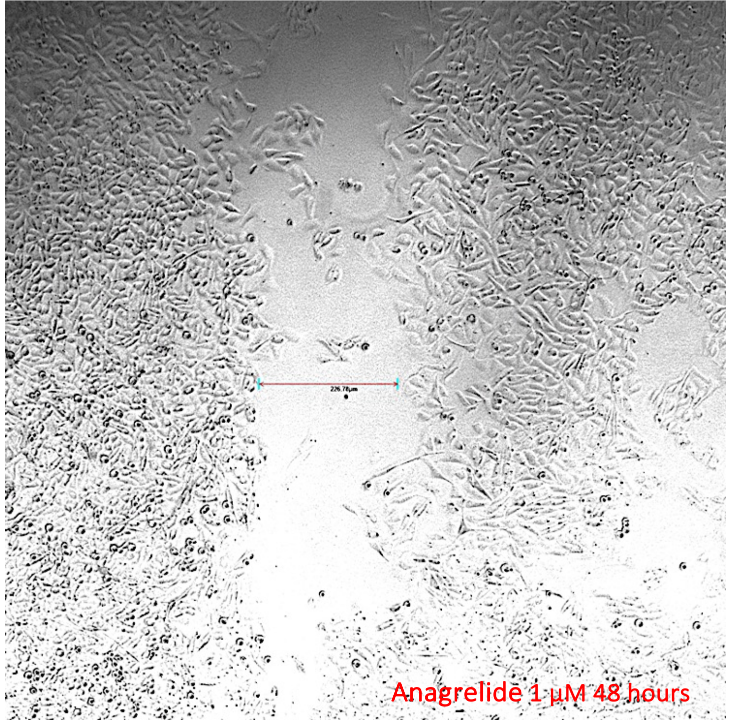

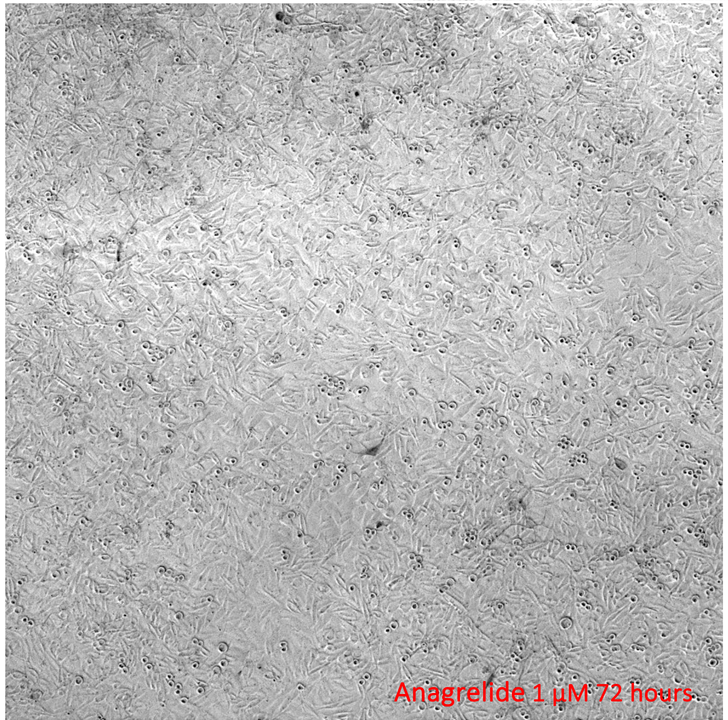

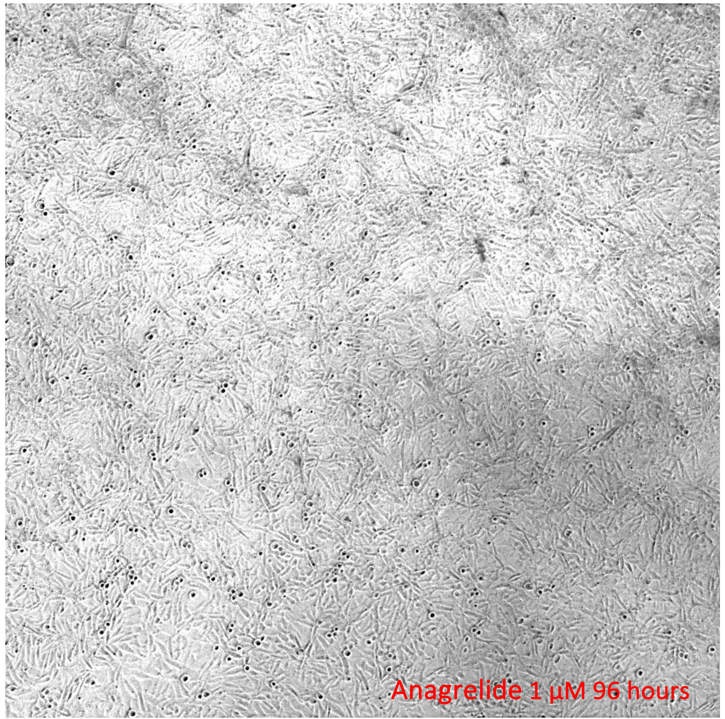

Anagrelide - 1 µM
Group

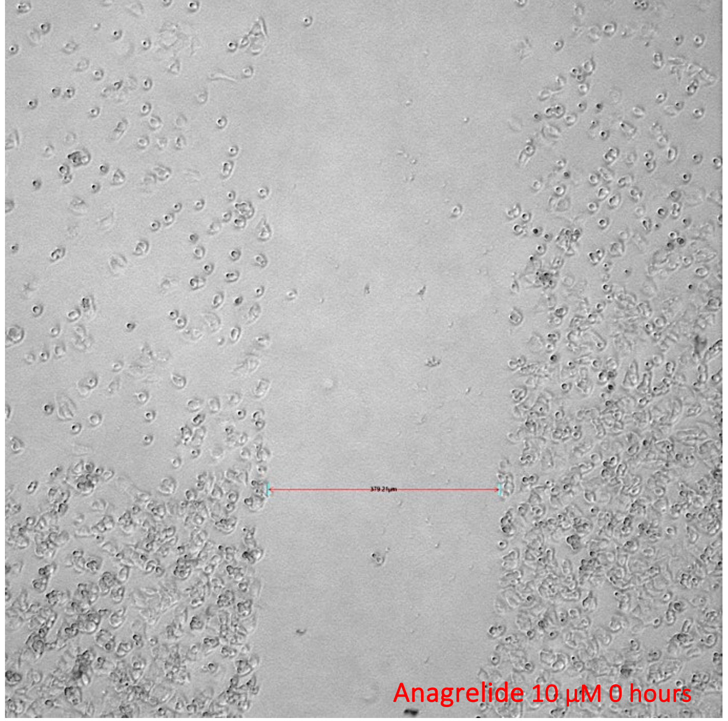

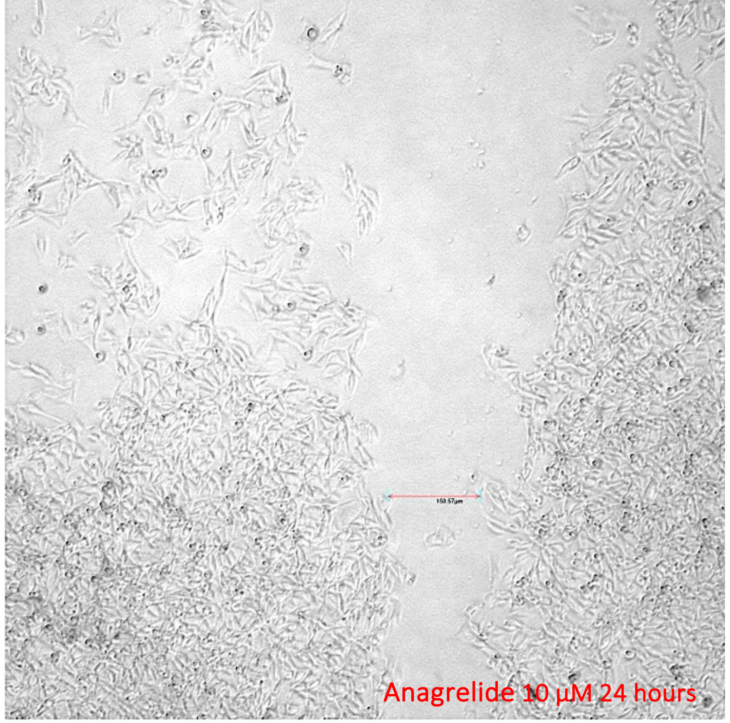

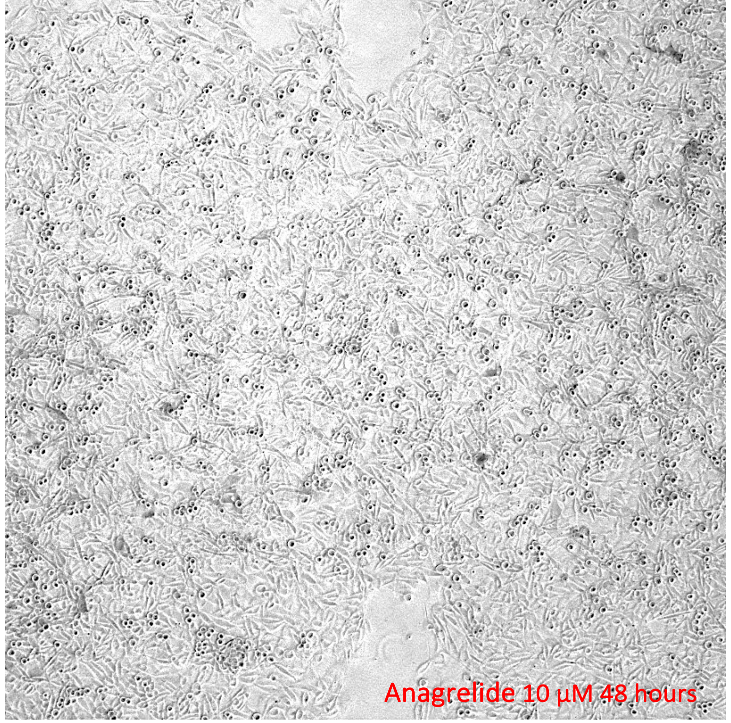

Anagrelide - 10 µM
Group

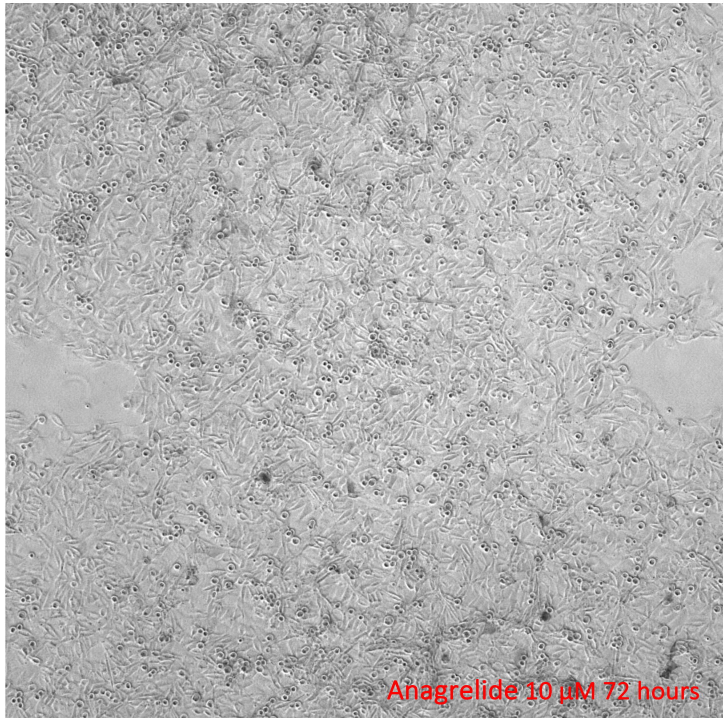

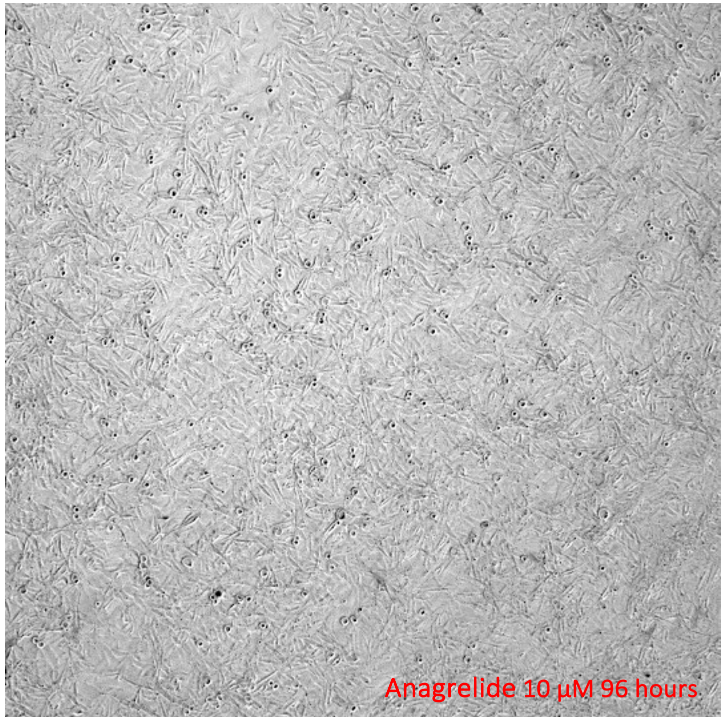

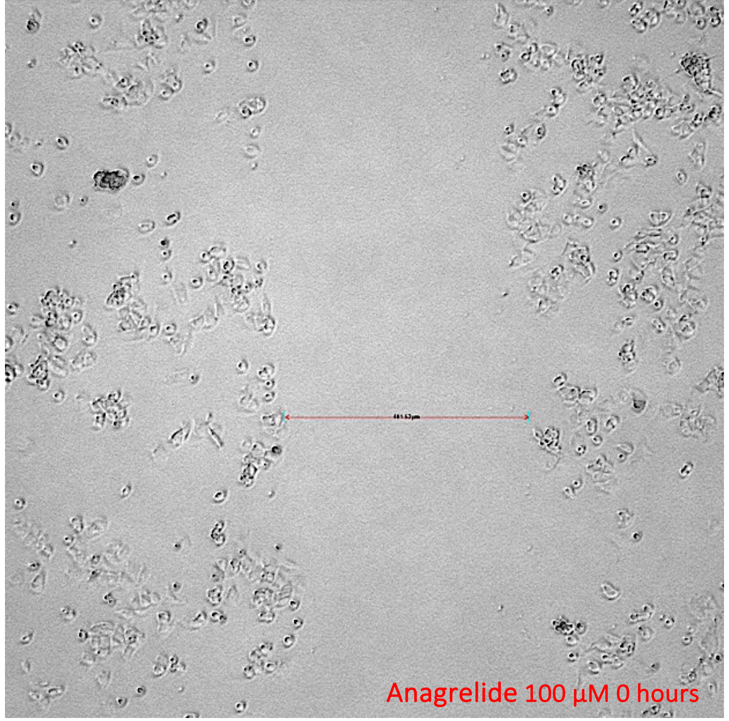

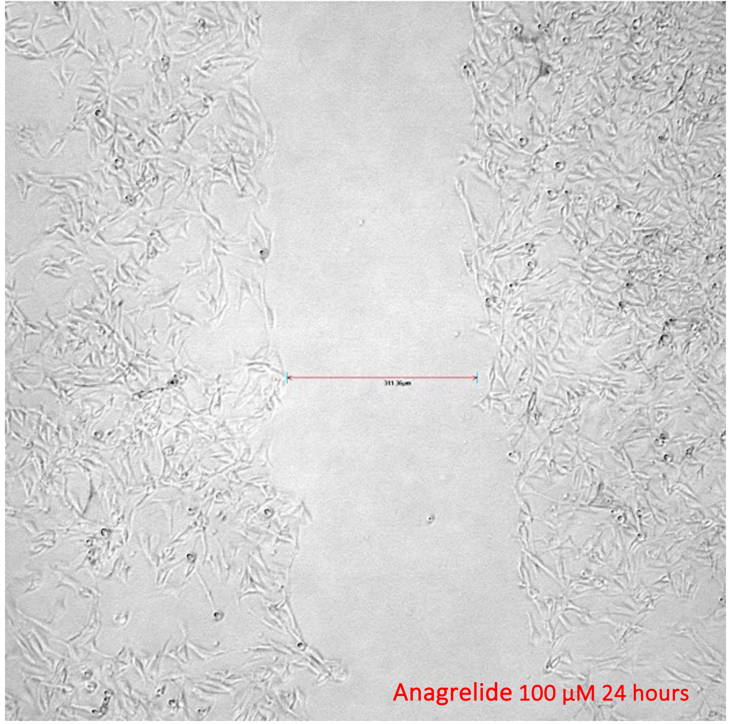

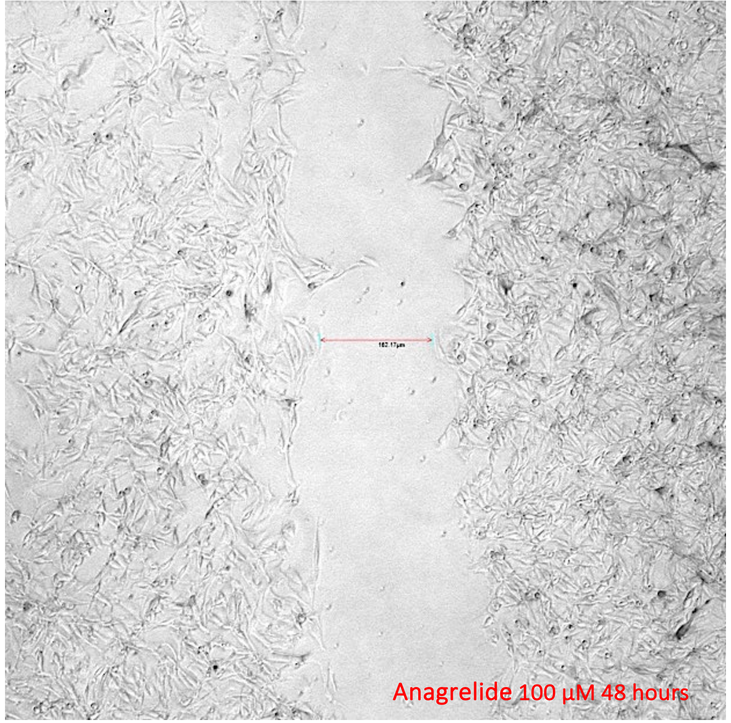

Anagrelide - 100 µM Group

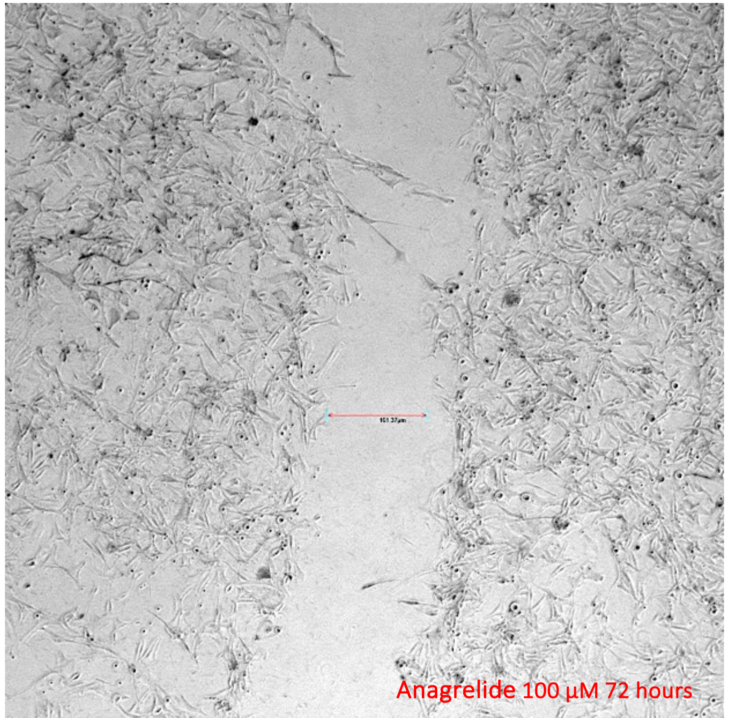

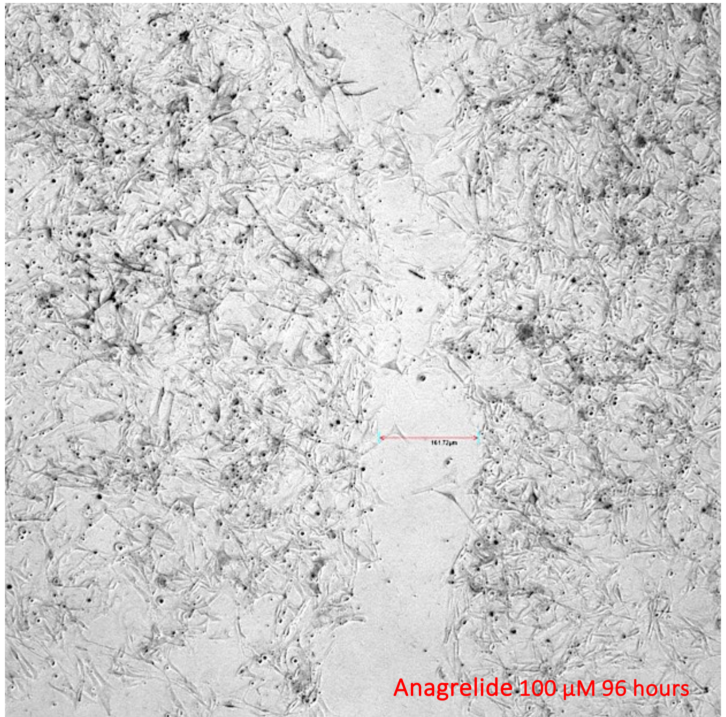

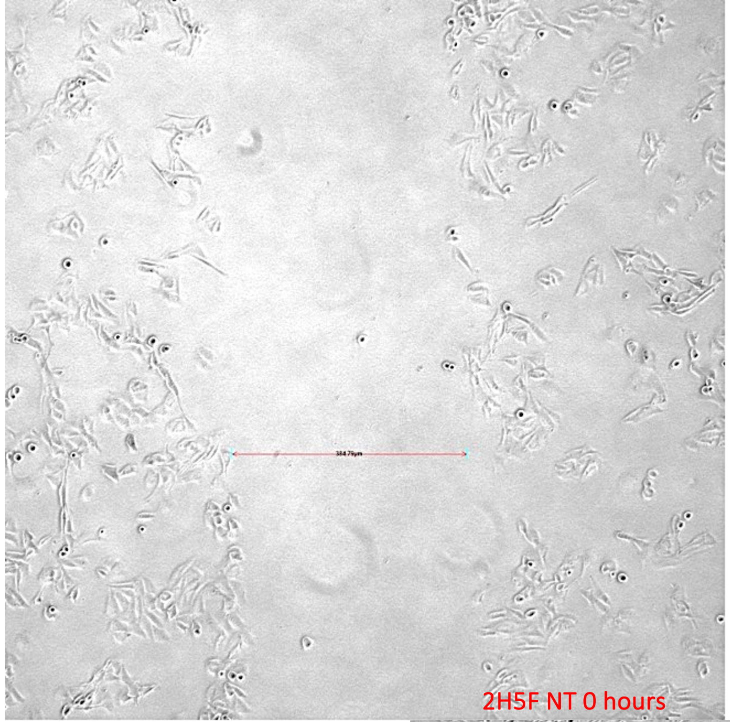

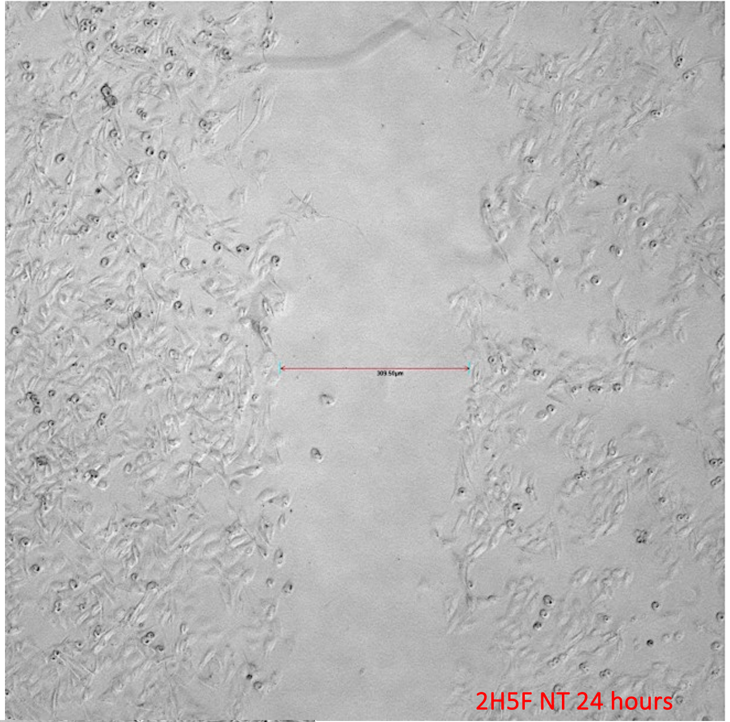

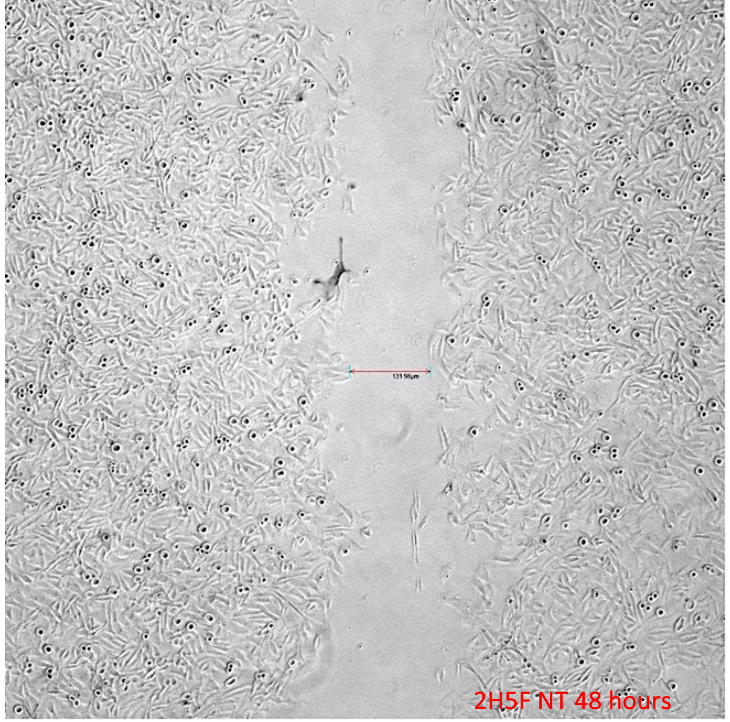

2H5F – NT Group

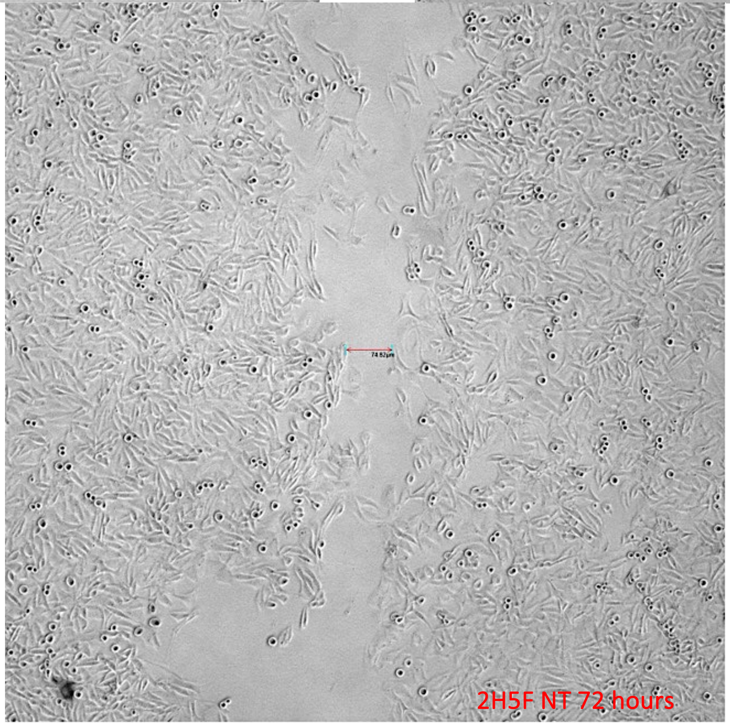

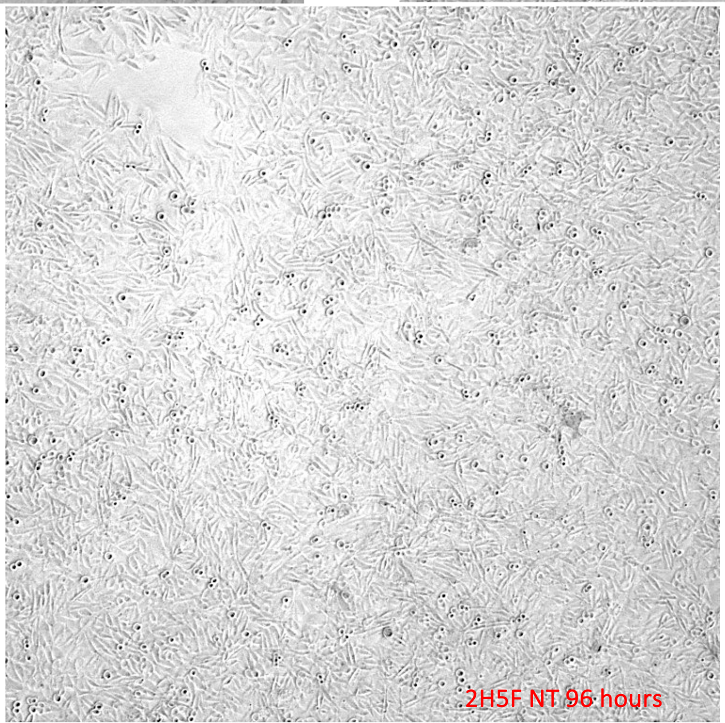

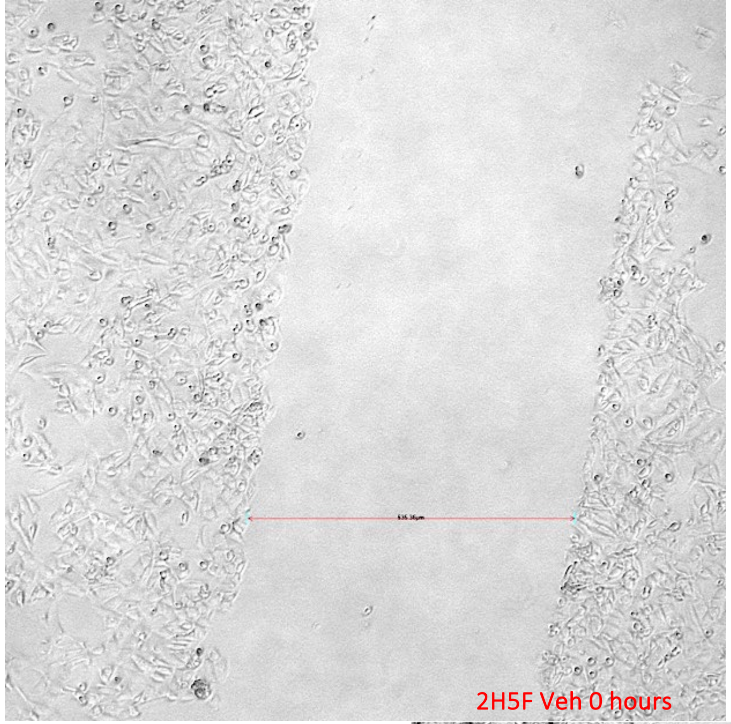

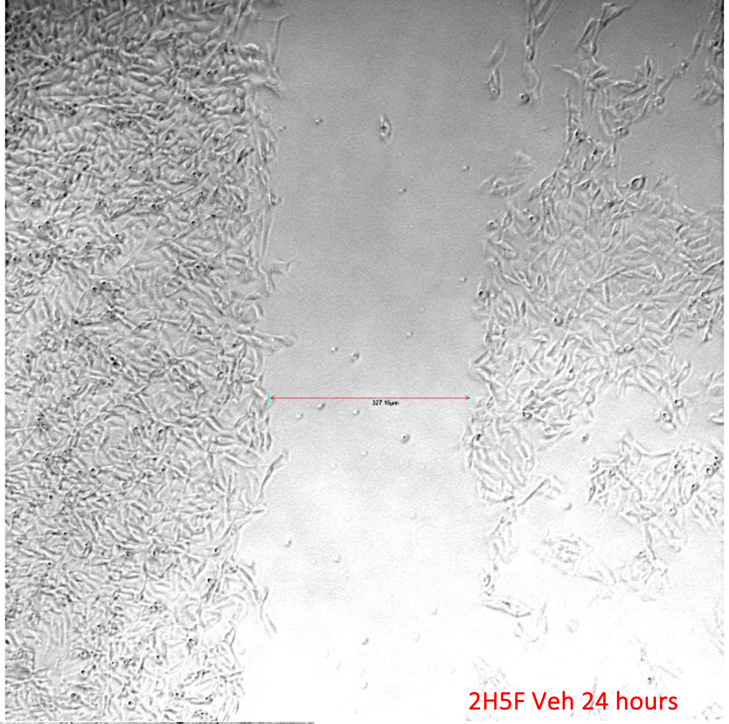

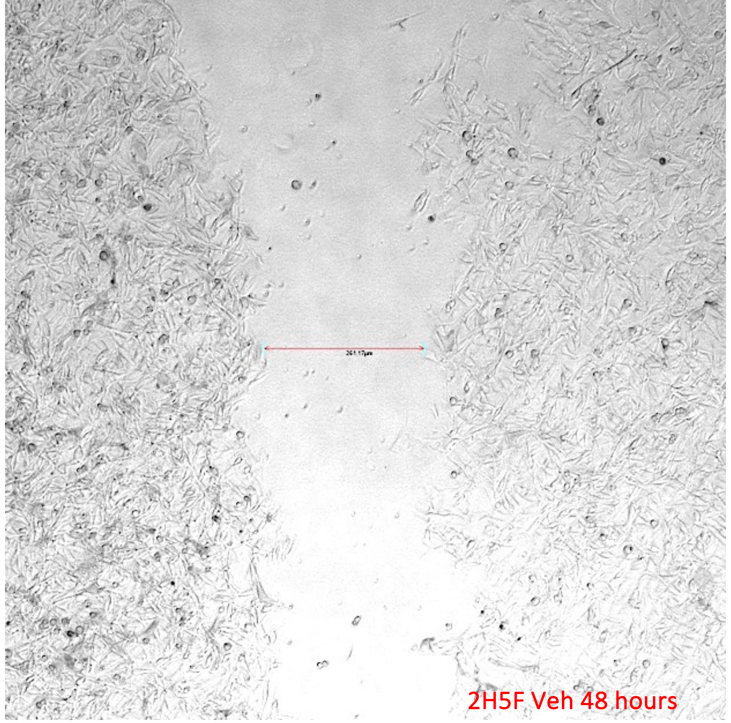

2H5F – Veh Group

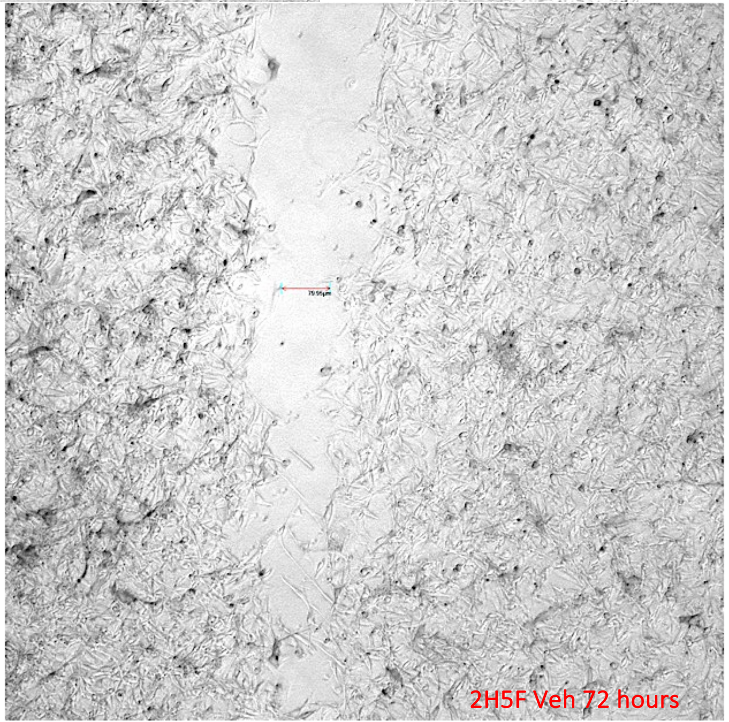

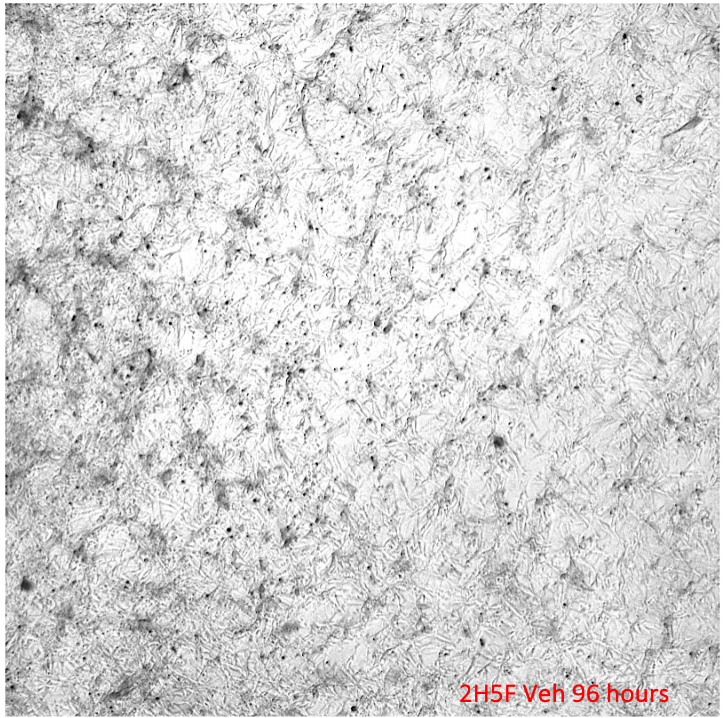

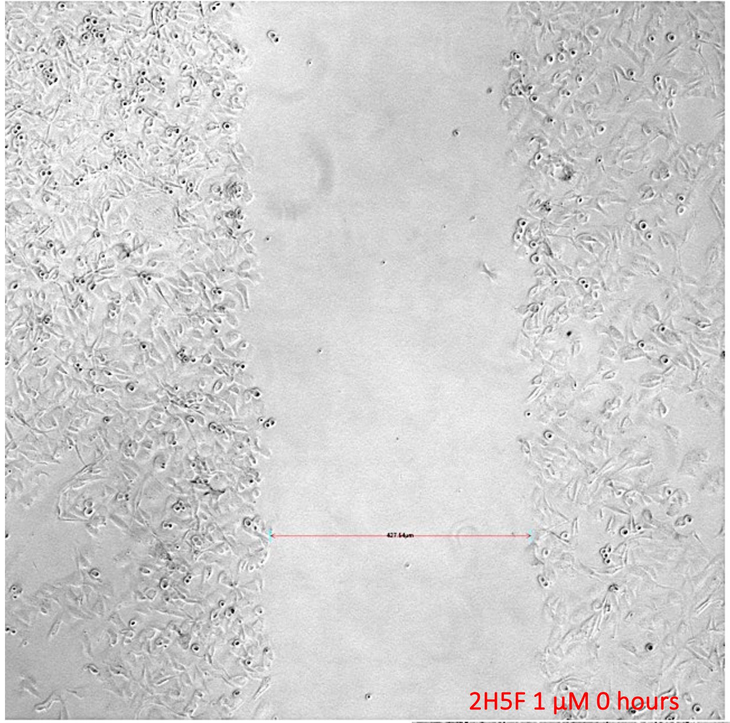

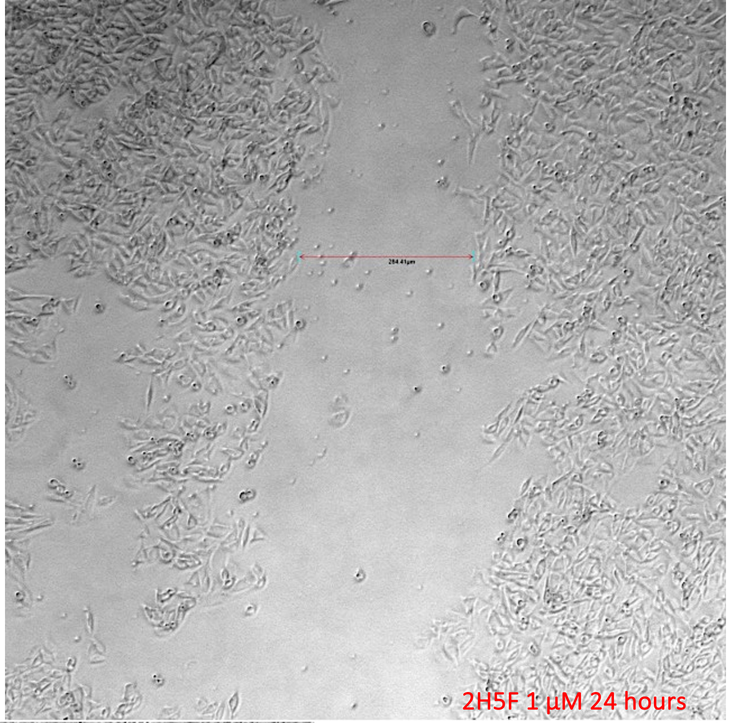

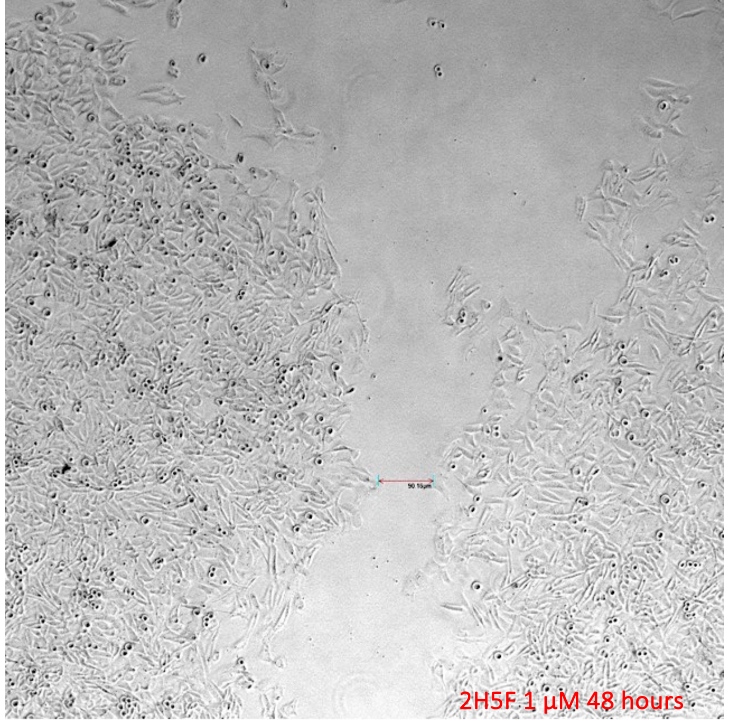

2H5F – 1 µM Group

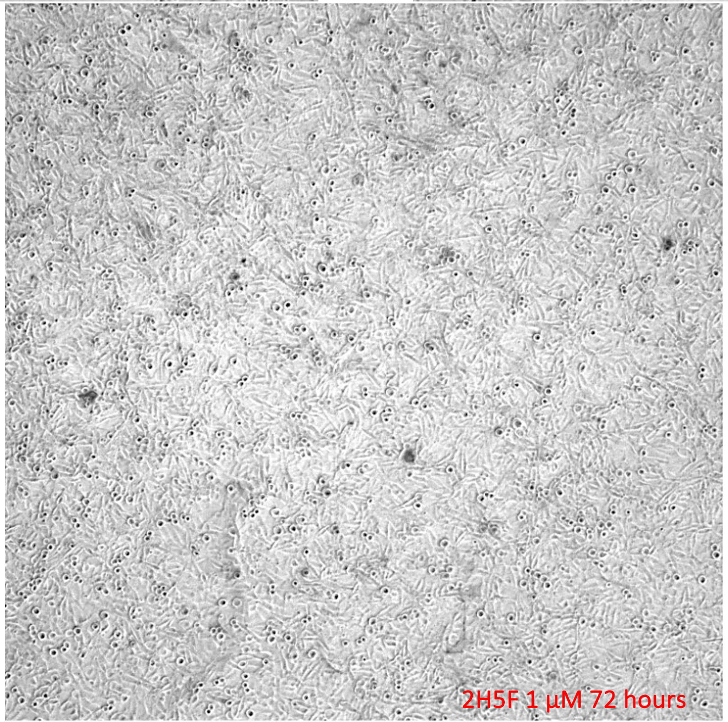

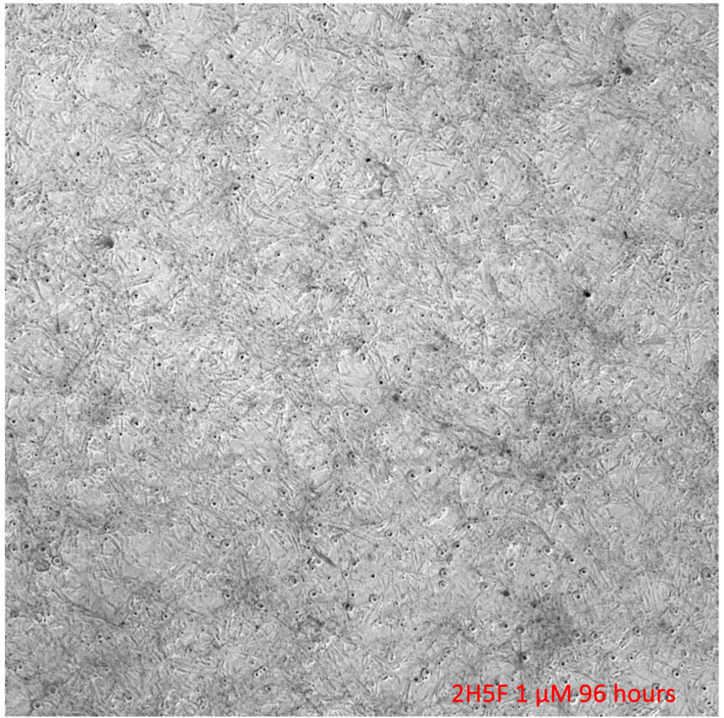

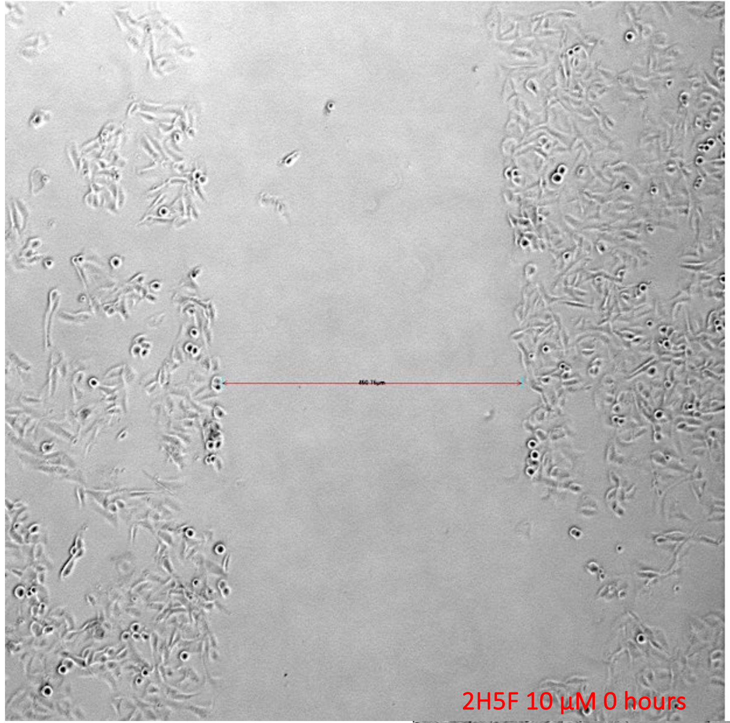

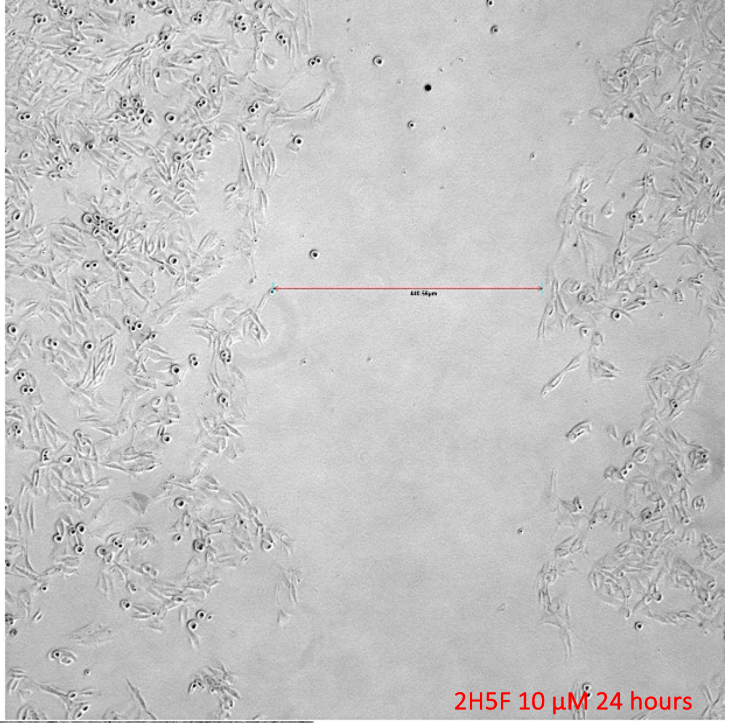

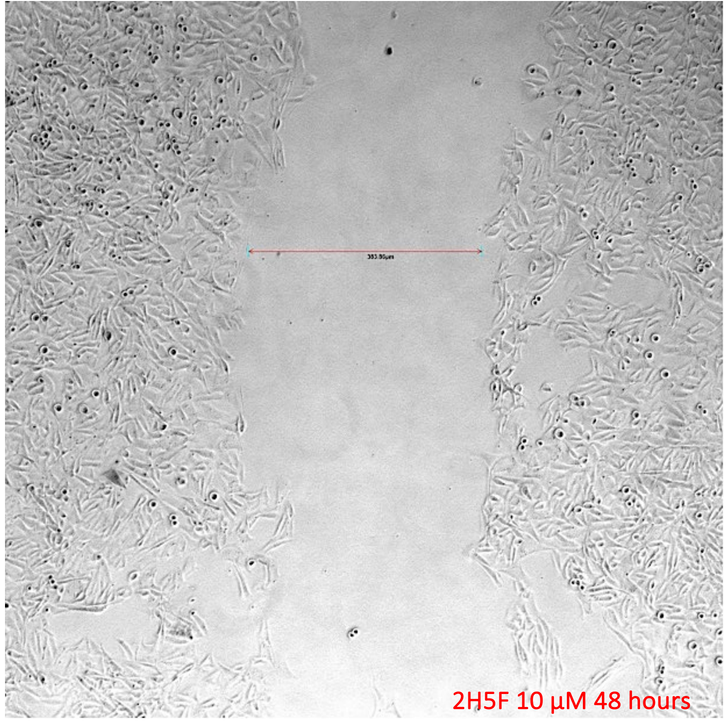

2H5F – 10 µM Group

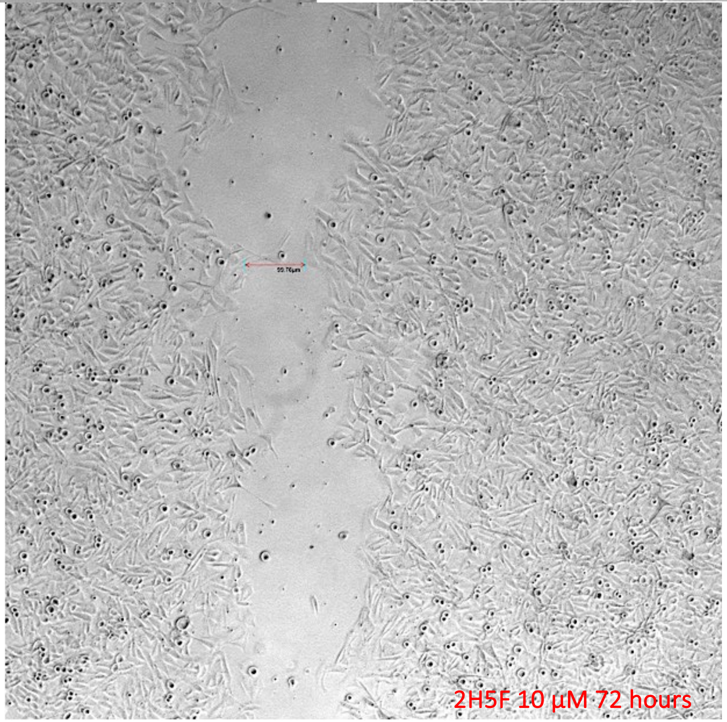

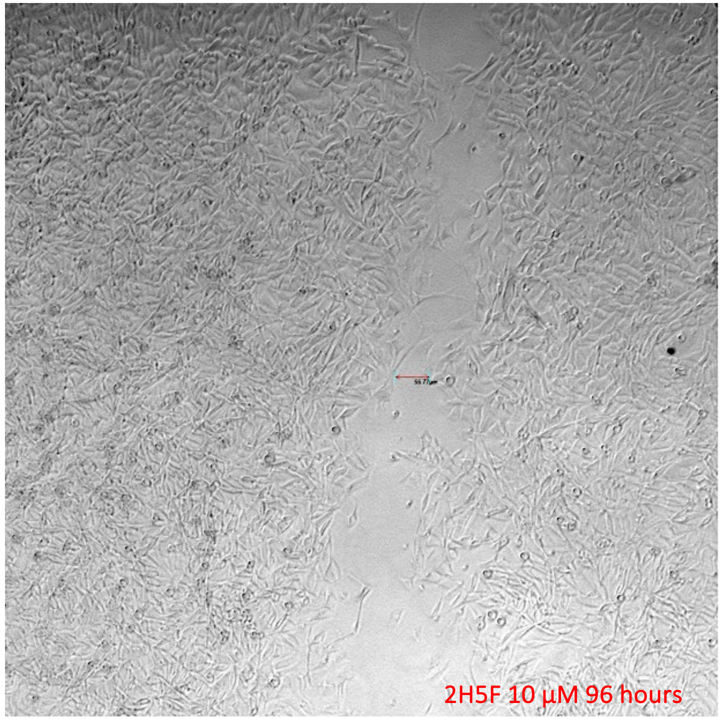

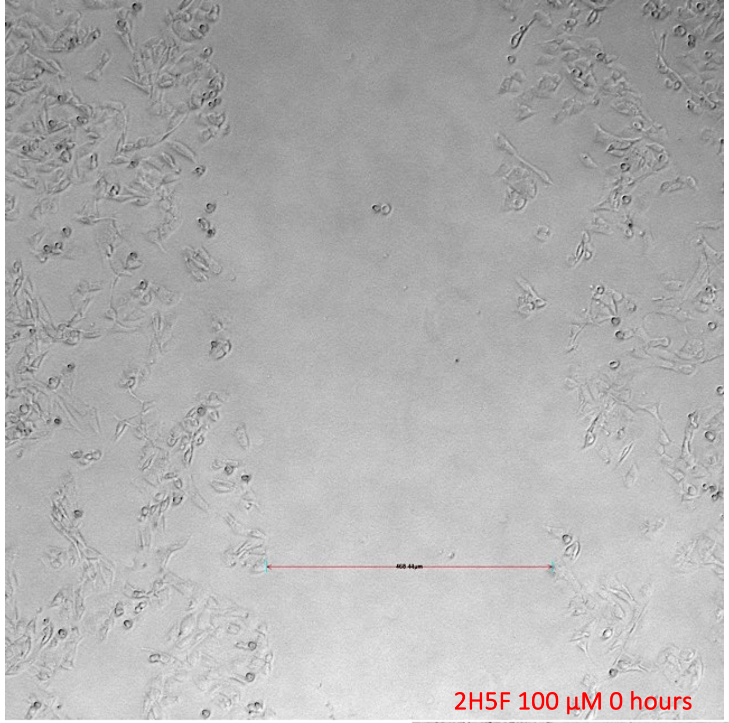

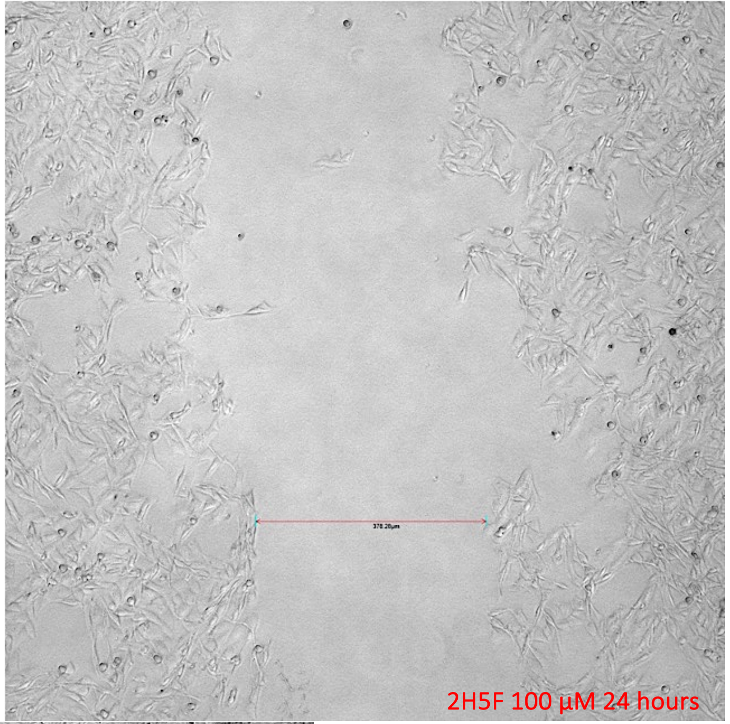

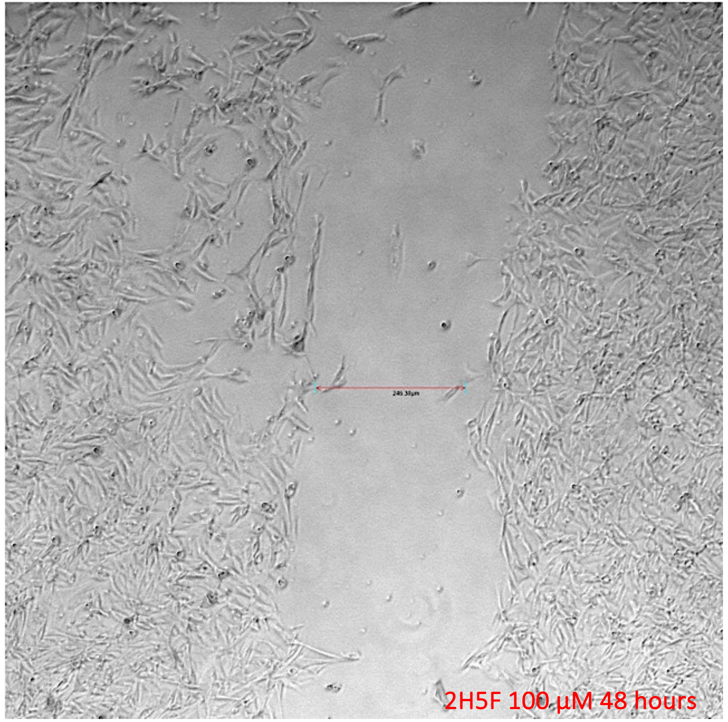

2H5F – 100 µM
Group

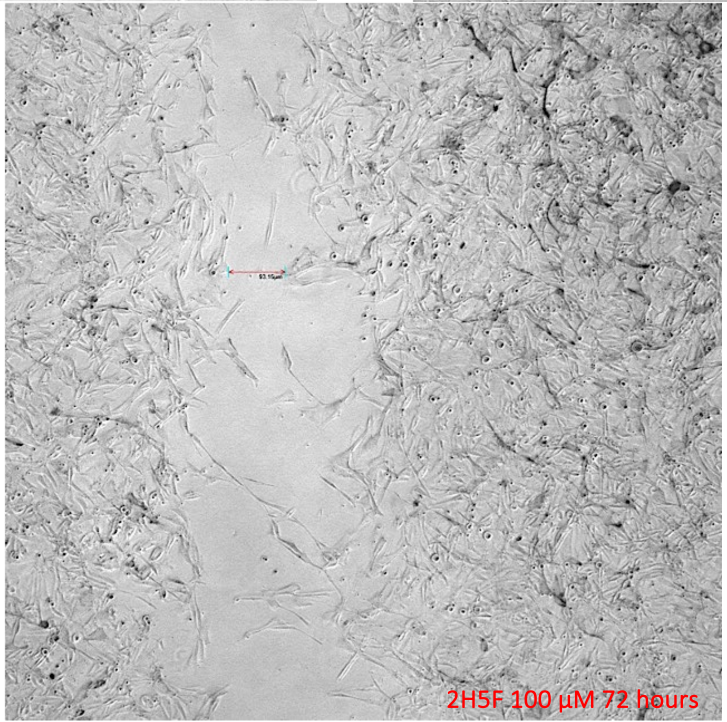

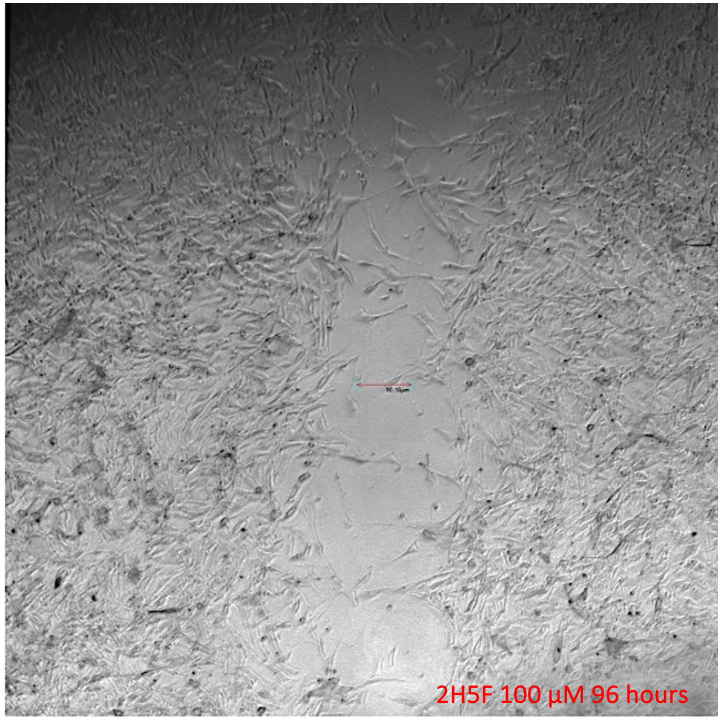

Supplement: Supplementary file 1 [file ijms-25-04811-s001.zip › ijms-2939066-supplementary.pdf]
